# Lower Limb Entheseal Variation in Modern Athletes and an Early Medieval Population: Implications for Activity Reconstruction

**DOI:** 10.1002/ajpa.70335

**Published:** 2026-08-18

**Authors:** Elena García‐Vargas, Jiří Macháček, Ángel Luis Parrado‐Gallego, Enrique Alcalá, Víctor Manuel Encinas‐Tobajas, Markus Bastir, Vladimír Sládek

**Affiliations:** ^1^ Department of Anthropology and Human Genetics Charles University Prague Czech Republic; ^2^ Department of Archaeology and Museology Masaryk University Brno Czech Republic; ^3^ Centro Nacional de Aceleradores Sevilla Spain; ^4^ Department of Applied Physics III, Engineering School University of Seville Seville Spain; ^5^ Musculoskeletal Unit, Radiology Department Hospital Universitario Virgen del Rocio Sevilla Spain; ^6^ Department of Pharmacology, Paediatric and Radiology, Area of Radiology and Physical Medicine University of Sevilla Sevilla Spain; ^7^ Paleoanthropology Group, Department of Paleobiology, Museo Nacional de Ciencias Naturales (MNCN‐CSIC) Calle José Gutiérrez Abascal Madrid Spain

**Keywords:** activity reconstruction, climbers, endurance runners, lower limb entheses, Pohansko, sprinters

## Abstract

**Objectives:**

Entheses are commonly used to infer past activity patterns, yet their interpretation is limited by the combined effects of mechanical loading and intrinsic biological factors. This study addresses this issue by integrating an Early Medieval population from Pohansko (Czechia; 9th–10th centuries CE) with a Spanish modern professional athlete reference sample characterised by well‐defined activity regimes.

**Materials and Methods:**

Lower limb entheseal areas of the *gluteus medius*, *gluteus maximus*, *vasti*, *gastrocnemius*, and *soleus* muscles were quantified in the archaeological individuals and in Modern Athletes (sprinters, endurance runners, climbers) using 3D modeling and the VERA protocol. Non‐adjusted and size‐adjusted data were analysed using univariate and multivariate approaches.

**Results:**

Modern Athletes exhibit significantly larger entheseal areas than Pohansko individuals for all muscles except the *soleus*, supporting the relationship between cumulative mechanical loading and entheseal development. Multivariate analyses differentiate both samples and distinguish locomotor strategies among athletes, with the *soleus* enthesis contributing most strongly to functional variation. Sexual dimorphism is consistent and male‐biased in athletes but variable and enthesis‐specific in the Pohansko sample.

**Discussion:**

Our results highlight the potential and limitations of entheseal areas for reconstructing behavioral and functional variation in past populations. In Modern Athletes, only the *soleus* enthesis distinguished climbers from runners, regardless of sex, but not sprinters from endurance runners. In Pohansko, it displayed marked female–male differences, consistent with a sex‐based division of labour. These findings suggest that lower limb entheses differ in their sensitivity to mechanical loading, with the *soleus* primarily reflecting locomotor loading rather than sex differences.

## 1 Introduction

Entheses (i.e., muscle, tendon, and ligament attachment sites) have been frequently used to reconstruct physical activity patterns from past populations (Beauval et al. 2005; Belcastro et al. 2020; Capasso et al. 1962; Castro et al. 2022; Dutour 1986; Foster et al. 2014; Havelková et al. 2011; Jurmain and Villotte 2010; Karakostis 2023; Karakostis, Jeffery, and Harvati 2019; Karakostis, Wallace, et al. 2019; Kubicka et al. 2022; Mariotti and Belcastro 2011; Robb 1994; Trinkaus et al. 1999; Villotte and Knüsel 2013; Weiss 2014). Traditionally, bioarchaeologists have turned to entheseal morphology to infer habitual behaviors, occupational activities, and division of labour (Beauval et al. 2005; Belcastro et al. 2020; Foster et al. 2014; Laurent et al. 2010; Mariotti and Belcastro 2011; Profico et al. 2025; Robb 1994; Villotte and Knüsel 2013). Because habitual behaviors generate biomechanical traces on the skeleton (Frost 1987; Larsen 1999; Ruff 1987; Ruff et al. 2006), physical activity reconstruction offers bioarchaeologists a powerful approach of inferring labour organization, mobility, and social differentiation (Godde et al. 2018; Havelková et al. 2013; Karakostis et al. 2017; Karakostis and Hotz 2024; Karakostis and Wallace 2023).

Entheses are dynamic, mechanosensitive structures rather than static skeletal marks. Foundational research established that entheses vary structurally according to their mechanical environment, forming either fibrous or fibrocartilaginous interfaces that affect the bone microarchitecture and load‐transfer properties (Benjamin et al. 2006). Fibrocartilaginous entheses exhibit a graded, four‐zone organization transitioning from tendon to bone, enabling progressive load dissipation and reducing mechanical stress concentrations (Apostolakos et al. 2014; Benjamin et al. 2006; Killian 2022; Lu and Thomopoulos 2013; Thomopoulos 2013). Experimental studies demonstrate that mechanical loading is essential for proper enthesis maturation, mineralization, and matrix organization, whereas unloading disrupts interface development (Killian 2022). Optogenetic work further showed that increased muscle contraction induces structural and molecular remodeling in the enthesis. These findings indicate that entheseal morphology reflects the integration of mechanical forces with biological responses, allowing us to interpret skeletal changes as potential markers of habitual activity when confounding factors are controlled.

However, entheseal morphology is multifactorial, not only responding to the mechanical loading of activity but also being shaped by age, sex, pathology, and body size (Henderson 2013; Moshrif et al. 2022; Schlecht 2012; Villotte et al. 2022; Villotte and Santos 2023; Zumwalt et al. 2006). Thus, behavioral inferences can remain unreliable, particularly when social or sexually dimorphic groups are compared in the absence of a rigorous comparative sample that incorporates controls for such confounding variables. Nonetheless, physical activity reconstruction provides a powerful approach of exploring social change, specifically in historical contexts where written sources are limited or biased. Hence, without well‐known comparative frameworks, it remains difficult to disentangle the relative contributions of mechanical loading and intrinsic biological factors when interpreting entheses in past populations, raising a fundamental challenge.

The analyses of entheseal areas are especially important when reconstructing the physical activity of societies experiencing profound political, social, and economic transformations such as the transitional populations from Early Medieval Central Europe. Archaeological and bioanthropological evidence from Great Moravian contexts, including Pohansko, indicates that patterns of activity, social organization, and subsistence strategies were highly structured yet not reducible to simple sex‐based divisions. Entheseal and cross‐sectional geometry analyses suggest moderate sexual dimorphism, with males generally exhibiting stronger limb bones but without extreme divergence, consistent with broader Holocene trends linking reduced mobility and agricultural lifeways to decreased sex differences in skeletal robusticity (Berner et al. 2018; Ruff 1987, 2018, 2019; Sládek et al. 2015; Sládek and Machácek 2017). At the same time, variation within sexes appears strongly conditioned by social status and economic roles: males engaged in agricultural labour show greater entheseal development than high‐status males, whereas high‐status females may exhibit higher robusticity than rural females, indicating that female labour remained physically demanding regardless of status (Havelková et al. 2011, 2013). Isotopic evidence from nearby Mikulčice further supports this pattern, showing that male activity varied with social position while female workloads remained consistently high (Kaupová et al. 2018).

In this context, archaeologically well‐documented populations provide a significant opportunity to evaluate how entheseal variation reflects structured behavioral differences rather than simple biological patterning. Archaeological data from Pohansko reinforce this complexity. The cemetery associated with the 9th–10th century church in the northeastern suburb likely represents a socially stratified but integrated community centred around an elite familia, including individuals of varying status rather than a strictly kin‐based group (Macháček 2010, 2019; Macháček et al. 2014, 2016; Macháček et al. 2019). Grave goods and settlement finds indicate a clear association between male elites and mounted warfare, with weapons, spurs, and horse equipment reflecting both status and habitual activity, while the symbolic use of such items in burials also signals ongoing processes of social stabilization and Christianisation (Klápště 2009, 2011; Macháček et al. 2016, 2021). The prominence of equestrian equipment suggests that horse riding formed a regular component of elite male behavior, consistent with broader cultural continuities from earlier Avar traditions in the Carpathian Basin (Bálint and Daim 1989; Bühler and Kirchengast 2022; Daim 1996; Pohl 1988; Macháček et al. 2024; Macháček 2026). However, not all individuals buried at Pohansko belonged to the elite, and lower‐status individuals, particularly women, were also present, with limited evidence for routine engagement in activities such as riding (Macháček et al. 2016; Macháček et al. 2021; Přichystalová 2012). Altogether, these data highlight that activity patterns in Pohansko were shaped by an interplay of sex, status, and cultural practices, underscoring the importance of using well‐contextualized comparative frameworks when interpreting entheseal variation in past populations.

To address this limitation, modern athlete populations offer a unique comparative framework for understanding skeletal responses to intensive, well‐defined mechanical loads and for isolating the effects of activity from confounding biological factors. Because the training regimes, movement patterns, and load exposures of Modern Athletes are well documented, they allow to observe how specific and sustained biomechanical demands influence musculoskeletal adaptation while controlling for variables such as sex, age, and body size (Davies et al. 2018; Hunter et al. 2023; Joyner et al. 2025; Ruff 2000). Endurance runners, sprinters, and climbers exhibit distinct lower limb loadings, producing corresponding differences in entheseal development and bone geometry (Carroll 2021; Hind et al. 2012; Joyner et al. 2025; Miller et al. 2024; Rooney et al. 2022) offering useful analogues for interpreting skeletal variation in past populations. The studies of Modern Athletes also clarify the role of biological sex in modulating musculoskeletal adaptation in terms of hormonal regulation, muscle architecture, and fiber composition influence and how individuals respond to identical mechanical stimuli, contribute to persistent sex‐related differences in strength, load distribution, and tissue remodeling (Davies et al. 2018; Hunter et al. 2023; Joyner et al. 2025; Ruff 2000). In fact, research on sprinters and endurance runners shows marked sex differences, with males typically displaying greater cortical thickness, muscle volume, and overall strength due to hormonal, size and shape factors (Davies et al. 2018; Hunter et al. 2023; Joyner et al. 2025; Ruff 2000). Although intensive training can partially narrow this gap, particularly in traits related to sustained effort, flexibility, and energy efficiency (Deaner et al. 2014; Hollander et al. 2021; Martins et al. 2023; Wickham et al. 2021; Wilks et al. 2009), sex‐specific differences persist: males remain generally stronger in short, high‐intensity activity, whereas females often excel in endurance stability and fatigue resistance (Mangine et al. 2014; Nuell et al. 2019). By contrast, climbers exhibit a considerably smaller sex‐based performance gap, likely because climbing relies on attributes such as relative strength, endurance, and flexibility, domains where female physiology may confer specific advantages (Carroll 2021; González‐Martín et al. 2025; Lamore et al. 2025). Moreover, injury patterns among sprinters, endurance runners and climbers further highlight sex‐specific biomechanical responses under similar training intensities, with females showing higher rates of bone stress injuries and males more tendon ruptures (Madsen et al. 2025; Popp et al. 2020). In sum, these athlete studies clarify the limits of bone plasticity and the role of sex in modulating skeletal adaptation to mechanical loads, providing an activity‐based comparative framework for interpreting past populations whose labour demands were persistent rather than specialized (Longman et al. 2020; Milks et al. 2019; Shaw and Stock 2013).

Despite these advances, a key limitation remains as there is a lack of studies directly linking activity patterns in modern populations with entheseal variation in archaeologically documented societies. Without such a bridge, interpretations of past behavior remain difficult to validate. Thus, this study addresses this gap by applying the Validated Entheses‐based Reconstruction of Activity (VERA) methodology, a three‐dimensional quantitative approach for the analysis of entheseal morphology that combines standardized measurements with a dedicated statistical framework for investigating activity‐related patterns (Karakostis 2023; Karakostis and Harvati 2021; Karakostis and Lorenzo 2016). The method has demonstrated high interobserver repeatability and has been experimentally validated in both human and non‐human studies (Karakostis et al. 2017; Karakostis, Jeffery, and Harvati 2019; Karakostis, Wallace, et al. 2019; Karakostis and Wallace 2023). Using the original VERA 1.0 protocol, we analyse lower limb entheses associated with muscles fundamental to locomotion and load bearing, that is, *gluteus medius*, *gluteus maximus*, *vasti*, *gastrocnemius*, and *soleus* (Bartlett et al. 2014; Ivanenko et al. 2004; Lenhart et al. 2014; Lin et al. 2011; Montgomery et al. 2016; Nicola and Jewison 2012; Thordarson 1997), in an Early Medieval Moravian population and a Modern Athlete sample.

By using Modern Athletes as a loading reference where activity patterns are well defined and variables such as sex, age, and body size can be explicitly accounted for, we are able to establish a framework with more clearly characterized loading regimes. This, in turn, provides a more robust baseline against which variation in archaeological populations can be interpreted. In addition, VERA has been evaluated through experimental studies and documented skeletal collections, supporting its utility for investigating activity‐related variation in muscle attachment sites (Karakostis et al. 2017; Karakostis, Wallace, et al. 2019; Karakostis and Hotz 2024; Karakostis and Wallace 2023). From this framework, we hypothesise that (1) Modern Athletes will display larger entheseal areas than Early Medieval individuals, reflecting the higher intensity and specificity of mechanical loading associated with athletic training, and that (2) Modern Athletes will exhibit lower levels of sexual dimorphism in entheseal morphology than Early Medieval individuals, due to the comparable training duration and intensity between Modern Athlete males and females relative to the sex‐differentiated activity patterns expected for Early Medieval populations.

## 2 Materials and Methods

### 2.1 Materials

This study is based on the analysis of two samples: the Pohansko Early Medieval population and Modern Athletes from Spain.

**FIGURE 1:**
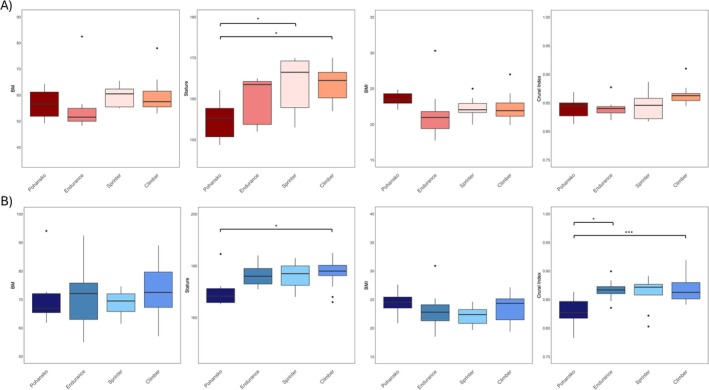
Boxplots of body mass (BM), stature, BMI, and crural index in (A) females and (B) males. Asterisks indicate significance: *p value < 0.05; **p value < 0.01; ***p value < 0.001.

The Pohansko sample (Table 1 and Figure 1) is composed of 30 adult males and 21 adult females that were excavated from the 9th century early medieval northeast suburb's second church Pohansko site (Czech Republic). The site has been dug and studied since its discovery in 1958, and it has been subjected to interdisciplinary research from 2008 until today (Sládek and Machácek 2017). The graves from which the studied individuals come started being dug in 2006, when the second church was discovered, and presented a good preservation, mostly because the site remained uninhabited after its decline in the 10th century. This study focuses on the analysis of the lower limbs of non‐pathological adult individuals that had been identified as males or females (Sládek and Machácek 2017) to accurately establish the comparison with the Modern Athletes' sample.

**TABLE 1:** Descriptive statistics for females and males, including the number of individuals, age, body mass (BM), stature, body mass index (BMI), femoral biomechanical length (FeBML), tibial biomechanical length (TiBML), and crural index.

Values (mean (± SD))	Female						Male					
	Pohansko (PF)	Endurance (EF)	Sprinter (SF)	Climber (CF)	Female total	Significant diff.	Pohansko (PM)	Endurance (EM)	Sprinter (SM)	Climber (CM)	Male total	Significant diff.
Number of Individual	21	8	8	11	48		30	13	9	15	67	
Age (in years)	37.59 (±10.792)	38.38 (±4.955)	24.13 (±4.581)	31.09 (±7.01)	34.77 (±10.283)	PF‐SF; EF‐SF	37.67 (±10.991)	34.08 (±8.588)	20.44 (±2.698)	33.93 (±6.63)	33.79 (±11.286)	PM‐SM; EM‐SM; SM‐CM
Body Mass (BM) (in kg)	56.58 (±5.500)	55.51 (±11.246)	59.71 (±3.998)	59.88 (±7.20)	57.03 (±6.836)	n.s.	70.34 (±7.563)	71.01 (±10.940)	68.50 (±4.220)	73.94 (±9.76)	70.19 (±8.010)	n.s.
Stature (in cm)	155.01 (±6.428)	160.00 (±5.855)	163.81 (±6.644)	163.55 (±4.54)	158.01 (±7.202)	PF‐SF; PF‐CF	169.83 (±6.769)	176.54 (±4.115)	176.17 (±4.835)	177.43 (±5.36)	172.60 (±6.665)	PM‐CM
BMI (in kg/m^2^)	23.52 (±1.779)	21.66 (±3.895)	22.289 (±1.553)	22.35 (±1.98)	22.84 (±2.426)	n.s.	24.34 (±1.477)	22.75 (±3.307)	22.10 (±1.615)	23.42 (±2.30)	23.55 (±2.262)	n.s.
Femur biomechanical length (FeBML) (in mm)	387.75 (±21.973)	355.39 (±21.013)	358.23 (±18.790)	405.20 (±19.12)	374.00 (±25.789)	n.s.	433.06 (±22.433)	387.36 (±16.533)	393.68 (±22.090)	443.24 (±18.30)	414.46 (±29.912)	n.s.
Tibia biomechanical length (TiBML) (in mm)	324.00 (±16.389)	334.99 (±23.600)	341.70 (±24.561)	350.01 (±16.10)	330.38 (±20.863)	n.s.	363.52 (±15.968)	378.70 (±13.581)	381.28 (±20.371)	385.39 (±17.87)	370.53 (±17.890)	n.s.
Crural index	0.84 (±0.020)	0.84 (±0.017)	0.85 (±0.025)	0.86 (±0.02)	0.84 (±0.020)	n.s.	0.83 (±0.027)	0.87 (±0.016)	0.86 (±0.029)	0.87 (±0.02)	0.85 (±0.027)	PM‐EM; PM‐CM

*Note:* Significant differences among samples are reported per sex.

The Modern Athlete sample (a total of 64 professional and semi‐professional male and female athletes) is composed of 17 sprinters (50 m, 100 m, 200 m and obstacles), 21 endurance runners (1000 m, 1400 m, marathoners and ultramarathoners) and 26 climbers (climbing wall, rock climbing, endurance climbing and speed climbing) (Table 1, Figure 1 and Supplementary Table S2). The participants were selected based on five requirements: (1) to be a male or female climber, sprinter or endurance runner, (2) to have no injuries or pathologies, (3) to be between 18 and 45 years old, (4) to be federated, with demonstrated experience (> 2 h/day or 4 days/week for at least a year) or competing at a high level (regional, national or international competitions) at the moment of the data collection and, (5) not to be pregnant, if female. To guarantee that the female participants were not pregnant at the moment of the data collection, on the day their CT scan was scheduled they had to take a pregnancy test at the Centro Nacional de Aceleradores (CNA) facilities provided by the principal investigator, and the result had to be negative to be able to proceed with the scanning.

All data collection procedures were reviewed and approved by the Ethics Committee of Charles University's Faculty of Sciences (approval numbers IRB Univerzita Karlova 2022/12, 2023/13 and 2024/03) and by the Scientific Commission of the Centro Nacional de Aceleradores (Seville), where the study was conducted. Prior to participation, all volunteers received a full briefing on the study objectives, analytical procedures, and associated risks, after which they provided written informed consent (World Medical Association 2013). Preceding the digital data acquisition, processing, and analysis, all data were anonymised in accordance with the principles of the Declaration of Helsinki (World Medical Association 2013).

### 2.2 Methods

To study the Pohansko individuals, the femur and tibia of each individual were scanned by using a Creaform HandySCAN 700 (Creaform Inc., Lévis, Québec, Canada), commonly used for the safe digitalization of fossils and remains (Friess 2012). The scanner works in concordance with the VX Software—EMS for 3D scanning, allowing us to precisely visualize the scanning process and determine the degree of precision of it. Only the right femur and tibia of each individual were analyzed since there is no significant asymmetry at the level of the lower limb (Auerbach and Ruff 2006). However, whenever the right femur or tibia was not available for the Pohansko individuals, the left one was used to perform the delineation and analyses of the entheses.

The Modern Athletes sample (Table 1, Figure 1, Supplementary Table S2) was collected at the CNA in Sevilla (Spain) between November 2023 and March 2025. Subjects were scanned in supine position from the top of their skull to the bottom of their feet. All image data were obtained with a 64‐row multidetector CT scanner (Siemens Biograph mCT, Erlangen, Germany) with the following parameters: slice width 0.6 mm; reconstruction increment 0.5 mm; collimation 64 × 0.6 mm; pitch 0.8; slice collimation 0.6 mm; voltage 120 kVp; reference tube current‐time 160 mAs; and rotation time 0.5 s. Routine matrix clinical images were reconstructed with B30 kernel to assess bone structures. The protocol was established by the radiologist of the study, Dr. Encinas, and performed by the CNA technician associated to this project, Ángel Parrado, to model as precisely as possible the cortical surface of the bones without exposing our participants to unnecessary radiation by using the Care Dose function. The Slicer software (Slicer 5.6.2 r32448/f10cd8c) was used to manually segment each one of the individuals' femur and tibia. First, by using the “Cropping module”, the data was cropped to be processed more efficiently, and then by using the “Segment Editor module”, the segment for each one of the bones was obtained (PerkLab Research 2018). Thresholding was performed manually. The resulting 3D models of the femur and tibia of the participants were then exported into an .obj format file.

**FIGURE 2:**
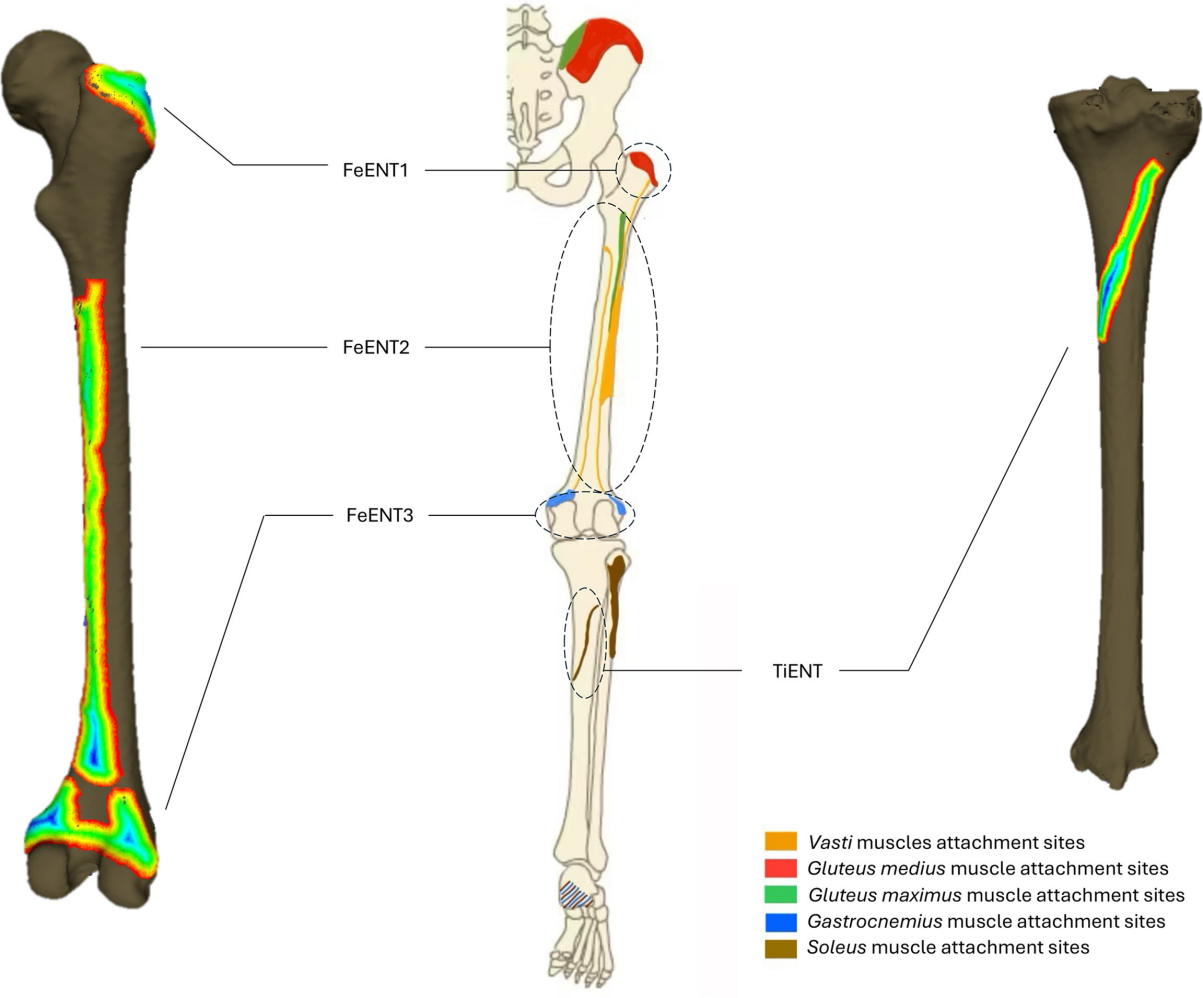
Overview of the entheses analyzed in the study. The figure presents three‐dimensional reconstructions of the femur and tibia models generated from a CT scan of the modern athlete CNA_010. The delineated areas incorporate the muscle attachment sites (vasti muscles, gluteus medius, gluteus maximus, gastrocnemius, and soleus), representing the enthesis points (FeENT1, FeENT2, FeENT3, and TiENT) examined in this research. These regions were used to quantify patterns of musculoskeletal variation within the sample.

The MeshLab software (MeshLab 64bit fp v2023.12) was used to apply the VERA protocol (Karakostis 2023; Karakostis and Harvati 2021; Karakostis and Lorenzo 2016) to identify the entheses of the *gluteus medius* (FeENT1), *gluteus maximus* and *vasti* (FeENT2), *gastrocnemius* (FeENT3), and *soleus* (TiENT) muscles (Figure 2 and Table 2) from all the individuals. After identifying and delineating the entheses (Figure 2), the detailed 3D surface area of each one of them was measured in mm^2^ by using the “Compute Geometric Measures” function provided by the MeshLab software itself. Furthermore, to understand the level of intraobserver error calculation that could result from the identification and delineation of the entheseal areas, the same procedure was repeated for a second time for 10 individuals (only the first measurements were used in the analyses). For each one of the entheses we calculated the maximum mean precision percentage error. Repeatability was high, with a precision error of 3.45%.

**TABLE 2:** Entheses examined in this study, including their abbreviations, associated bone, corresponding muscle, proximal and distal muscle attachments, primary movement involved, and relevant references reporting similar analyses or anatomical descriptions.

Entheses abbreviation	Bone	Muscle	Proximal attachment	Distal attachment	Movement	References
FeENT1	Femur	Gluteus medius	External surface of ilium between anterior and posterior gluteal lines	Lateral surface of greater trochanter of femur	Abduct and medially rotate thigh; keep pelvis level when ipsilateral limb is weight‐bearing and advance opposite (unsupported) side during its swing phase.	Mariotti et al. 2004; Havelková, Hladík, Velemínský 2012; Lieverse et al. 2013; Takigawa 2014
FeENT2	Femur	Gluteus maximus	Ilium posterior to posterior gluteal line; dorsal surface of sacrum and coccyx; sacrotuberous ligament	Most fibers end in iliotibial tract, which inserts into lateral condyle of tibia; some fi bers insert on gluteal tuberosity	Extends thigh (especially from flexed position) and assists in its lateral rotation; steadies thigh and assists in rising from sitting position.	Robb 1994; Al‐Oumaoui et al. 2004; Lieverse et al. 2013; Takigawa 2014
		Vasti muscles	Greater trochanter and lateral lip of linea aspera of femur; intertrochanteric line and medial lip of linea aspera of femur; anterior and lateral surfaces of shaft of femur	Via common tendinous (quadriceps tendon) and independent attachments to base of patella; indirectly via patellar ligament to tibial tuberosity; medial and lateral vasti also attach to tibia and patella via aponeuroses (medial and lateral patellar retinacula)	Extend leg at knee joint; rectus femoris also steadies hip joint and helps iliopsoas flex thigh	Robb 1994; Al‐Oumaoui et al. 2004; Lieverse et al. 2013; Takigawa 2014
FeENT3	Femur	Gastrocnemius	Lateral head: lateral aspect of lateral condyle of femur Medial head: popliteal surface of femur; superior to medial condyle	Posterior surface of calcaneus via calcaneal tendon	Plantar flexes ankle when knee is extended; raises heel during walking; flexes leg at knee joint.	Al‐Oumaoui et al. 2004
TiENT	Tibia	Soleus	Posterior aspect of head and superior quarter of posterior surface of fi bula; soleal line and middle third of medial border of tibia; and tendinous arch extending between the bony attachments	Posterior surface of calcaneus via calcaneal tendon	Plantar flexes ankle when knee is extended; raises heel during walking; flexes leg at knee joint.	Al‐Oumaoui et al. 2004; Lieverse et al. 2013; Takigawa 2014

After obtaining the surface area of the entheses, they were adjusted to size as previous studies have demonstrated that even though habitual activity correlates with entheseal areas (Karakostis, Wallace, et al. 2019; Karakostis and Harvati 2021; Kunze et al. 2024; Schlecht 2012), they are also affected by bone and body size, age, and pathologies for which it is important to control for (Moshrif et al. 2022; Ruff 2003; Villotte and Santos 2023). To reduce the impact of size, each entheseal area was adjusted to size:

(1) $$\mathrm{ENT}\_\mathrm{BM}\times \mathrm{BML}=\frac{\mathrm{ENT}}{\mathrm{BM}\times \mathrm{BML}}\times 10^{4}$$

where ENT_BM×BML is the adjusted to size entheseal area, ENT is the unadjusted entheseal area, BM is body mass, and BML is the biomechanical length of the femur or the tibia following Trinkaus et al. (1999).

The Body Mass data from the Modern Athletes was obtained directly at the CNA the day their CT scans were performed as they also had to be measured and weighted. Regarding the Pohansko individuals, their BM was estimated using a morphometric approach and population specific femoral head equations derived for Central European Early Medieval period samples (Sládek and Machácek 2017). This size adjustment technique was preferred because though body mass values differ in nature between samples, with measured values used for Modern Athletes and osteological estimates used for Pohansko individuals, the relatively lean body composition of athletes may reduce discrepancies in scaling relationships. Additionally, by using the biomechanical length of the bones it is possible to also balance this limitation. Lastly, this size‐adjustment method was preferred, contrary to for example geometric mean, as it allows direct comparisons of overall entheseal size between the Pohansko and Modern Athlete samples, which constitutes one of the primary objectives of the present study. Nonetheless, other size‐adjustment techniques (i.e., body mass) were also applied (Supplementary Figures S1 and S2) and yielded consistent results.

Only those individuals that presented values for all the entheses studied in this project were used. The samples were described and compared based on their weight, height, femoral biomechanical length and tibial biomechanical length (Trinkaus et al. 1999). To evaluate the influence of body size, the relationships between femoral and tibial entheseal surface areas and body mass, stature, and biomechanical bone length were assessed using Pearson correlation analysis. These analyses allow to better understand the proportion of variability that could be explained by each one of these variables, and hence to control for all those factors that can have a potential impact on the size and shape of entheses not being related to physical activity. To understand if the correlations between the samples were significantly different among each other, an Analysis of Variance (ANOVA) was conducted followed by a post hoc Tukey's range test to interpret the statistical difference between them. Normality was assessed using the Shapiro–Wilk test. Moreover, to quantify the level of sexual dimorphism among females and males from the same sample, a sexual dimorphism calculation (SD) was implemented by using the following equation (Auerbach and Ruff 2006; Sládek et al. 2017):

(2) $$\frac{M-F}{\left(M+F\right)/2}\times 100,$$

where *M* is the males' mean for the enthesis and *F* is the females' mean for the enthesis.

After verifying all assumptions, we used a correlation‐based PCA on unadjusted entheseal areas to identify multivariate patterns without prior group classification (Abdi and Williams 2010; Field 2013; Hammer and Harper 2024; Karakostis, Jeffery, and Harvati 2019; Zelditch et al. 2004). To get a better understanding of the PCA results, a Multivariate Analysis of Variance (MANOVA) and a Canonical Variate Analysis (CVA) were carried out. The MANOVA test allowed to test for differences in variance among our three samples while the CVA projects multivariate data onto a coordinate system that maximizes the separation between given groups and identifies the linear combination of parameters providing a maximal separation of the samples analyzed (Field 2013; Hammer and Harper 2024; Zelditch et al. 2004). Lastly, we also applied a PCA to entheseal areas expressed as relative proportions to control for differences in overall size and isolate meaningful patterns in their compositional variation. The effects of age, height, activity and sex, and their potential interactions on relative entheseal proportions were assessed using multivariate Procrustes analyses of variance with residual randomization (1000 permutations). The interaction between age and activity was also assessed to determine whether age‐related variation differed among activity groups. Where significant associations were detected, correlations between the corresponding principal component (PC) scores and the predictor variable were examined to identify the principal axes underlying the observed patterns.

Additionally, individual boxplots of unadjusted and size‐adjusted entheses were used to visualize sample distribution and significance. ANOVA and Tukey's range test were used to test whether groups differ in unadjusted and adjusted entheseal surface area between groups.

All the statistical analyses and figures were performed in the R software (R Core Team 2025) using the “readxl”, “mice”, “dplyr”, “lmodel2”, “MASS”, “candisc”, “tidyr” and “ggplot2” packages.

## 3 Results

### 3.1 Samples Comparisons

Table 1 summarizes differences in body proportions between the Pohansko individuals and the athlete groups. Among females, Pohansko individuals are significantly shorter than both sprinters and climbers, while sprinters are significantly younger than both Pohansko females and endurance runners. Among males, Pohansko individuals are significantly shorter than climbers, and sprinters are significantly younger than Pohansko, endurance, and climber males. Pohansko males also show significantly lower crural index values than endurance runners and climbers. When sexes are pooled (Supplementary Table S2), sprinters are significantly younger than most other groups. Across all samples, males exhibit significantly greater stature and body mass than females, although BMI does not differ significantly among any of the sex‐specific or pooled groups. Athlete males have significantly longer femoral and tibial biomechanical lengths than all female groups. Pohansko males also have significantly longer femora than endurance and Pohansko females, and significantly longer tibiae than Pohansko females. Additionally, Pohansko males exhibit significantly lower crural index values than male endurance runners, male climbers, and female climbers.

Tables 3 and 4 present correlations between body dimensions (body mass, stature, femoral and tibial biomechanical lengths) and unadjusted entheseal areas for females and males respectively. Stature shows strong and consistent correlations with femoral and tibial biomechanical lengths across all groups, with no significant differences in regression slopes. Body mass correlates with the *gluteus medius* and *gastrocnemius* entheseal areas (FeENT1 and FeENT3 respectively) among Pohansko, endurance runner, and climber males, although slopes do not differ significantly between samples. Femoral and tibial biomechanical lengths are strongly correlated in every group, and their relationships with the *gluteus medius*, *gastrocnemius*, and *soleus* entheses (FeENT1, FeENT3, TiENT) vary among samples but do not differ significantly in slope.

**TABLE 3:** Summary of least squares regression analyses showing the relationship between each predictor variable (BM, Stature, FeBML and TiBML) and the corresponding response variables (BM, Stature, FeBML, TiBML, FeENT1, FeENT2, FeENT3 and TiENT) among females.

Regressions analyzed	Pohansko (*n* = 6)			Endurance (*n* = 8)			Sprinter (*n* = 8)			Climber (*n* = 11)		
	Slope	*r* squared	*p*‐value	Slope	*r* squared	*p*‐value	Slope	*r* squared	*p*‐value	Slope	*r* squared	*p*‐value
BM												
Stature	0.836	0.96	< 0.001	n.s.	0.12	0.197	n.s.	0.35	0.062	0.441	0.45	0.012
FeBML	3.841	0.84	0.005	n.s.	< 0.01	0.449	n.s.	0.21	0.127	1.904	0.39	0.019
TiBML	2.537	0.88	0.003	n.s.	< 0.01	0.453	n.s.	0.22	0.118	1.599	0.40	0.019
FeENT1	n.s.	0.24	0.161	n.s.	0.30	0.078	n.s.	0.25	0.102	n.s.	0.08	0.255
FeENT2	n.s.	0.05	0.335	n.s.	0.08	0.254	n.s.	0.13	0.192	n.s.	< 0.01	0.441
FeENT3	n.s.	0.33	0.115	n.s.	0.03	0.354	n.s.	0.05	0.289	69.771	0.56	0.017
TiENT	n.s.	< 0.01	0.495	n.s.	0.07	0.269	n.s.	0.04	0.309	n.s.	0.02	0.359
Stature												
BM	1.196	0.96	< 0.001	n.s.	0.12	0.197	n.s.	0.35	0.062	2.268	0.45	0.012
FeBML	4.885	0.88	0.003	3.939	0.84	0.001	2.841	0.83	0.003	4.488	0.77	< 0.001
TiBML	3.153	0.93	0.001	4.285	0.80	0.001	3.953	0.76	0.002	3.665	0.86	< 0.001
FeENT1	n.s.	0.36	0.104	n.s.	0.14	0.180	n.s.	0.02	0.362	n.s.	0.01	0.382
FeENT2	n.s.	0.02	0.392	n.s.	0.33	0.070	n.s.	< 0.01	0.489	n.s.	0.08	0.196
FeENT3	n.s.	0.27	0.144	n.s.	0.17	0.158	n.s.	0.22	0.123	n.s.	0.17	0.102
TiENT	n.s.	0.01	0.422	n.s.	0.25	0.101	n.s.	0.05	0.289	n.s.	0.08	0.202
FeBML												
BM	0.260	0.84	0.005	n.s.	< 0.01	0.449	n.s.	0.21	0.127	0.525	0.39	0.019
Stature	0.205	0.88	0.003	0.254	0.84	0.001	0.352	0.83	0.001	0.223	0.77	< 0.001
TiBML	0.649	0.86	< 0.001	1.062	0.95	< 0.001	1.332	0.93	< 0.001	0.883	0.84	< 0.001
FeENT1	14.208	0.51	0.054	14.979	0.39	0.049	n.s.	0.17	0.152	n.s.	0.16	0.146
FeENT2	n.s.	0.13	0.245	n.s.	0.25	0.105	n.s.	< 0.01	0.435	n.s.	0.28	0.070
FeENT3	n.s.	0.47	0.067	n.s.	0.35	0.062	n.s.	0.21	0.128	n.s.	0.12	0.182
TiENT	n.s.	< 0.01	0.474	n.s.	0.30	0.079	n.s.	0.02	0.359	n.s.	< 0.01	0.461
TiBML												
BM	0.39	0.88	0.003	n.s.	< 0.01	0.453	n.s.	0.22	0.118	0.625	0.40	0.019
Stature	0.32	0.93	0.001	0.23	0.80	0.001	0.25	0.76	0.002	0.273	0.86	< 0.001
FeBML	1.54	0.86	0.004	0.94	0.95	< 0.001	0.75	0.93	< 0.001	1.190	0.83	< 0.001
FeENT1	n.s.	0.36	0.104	n.s.	0.29	0.085	n.s.	0.10	0.219	n.s.	0.13	0.139
FeENT2	n.s.	< 0.01	0.472	n.s.	0.21	0.129	n.s.	0.02	0.386	n.s.	0.01	0.371
FeENT3	n.s.	0.18	0.198	25.97	0.39	0.048	n.s.	0.08	0.245	n.s.	0.14	0.127
TiENT	n.s.	0.09	0.286	n.s.	0.17	0.158	n.s.	0.05	0.306	n.s.	0.03	0.341

*Note:* Reported are the estimated slope, *r* square, and *p*‐value for each regression.

**TABLE 4:** Summary of least squares regression analyses showing the relationship between each predictor variable (BM, Stature, FeBML and TiBML) and the corresponding response variables (BM, Stature, FeBML, TiBML, FeENT1, FeENT2, FeENT3 and TiENT) among males.

Regressions analyzed	Pohansko (*n* = 7)			Endurance (*n* = 13)			Sprinter (*n* = 9)			Climber (*n* = 15)		
	Slope	*r* squared	*p*‐value	Slope	*r* squared	*p*‐value	Slope	*r* squared	*p*‐value	Slope	*r* squared	*p*‐value
BM												
Stature	0.545	0.71	0.009	n.s.	0.18	0.076	n.s.	0.05	0.283	0.406	0.49	0.002
FeBML	2.363	0.78	0.004	n.s.	0.04	0.262	n.s.	0.06	0.261	1.077	0.27	0.023
TiBML	1.098	0.70	0.009	n.s.	0.10	0.152	n.s.	< 0.01	0.473	1.380	0.48	0.002
FeENT1	61.841	0.45	0.048	44.243	0.44	0.007	n.s.	0.07	0.247	n.s.	0.18	0.068
FeENT2	205.289	0.56	0.026	n.s.	0.05	0.239	n.s.	0.21	0.107	127.256	0.32	0.018
FeENT3	n.s.	0.04	0.343	n.s.	0.01	0.402	n.s.	0.04	0.313	48.662	0.41	0.007
TiENT	n.s.	0.13	0.211	n.s.	< 0.01	0.442	n.s.	0.13	0.172	n.s.	0.16	0.075
Stature												
BM	n.s.	0.71	0.009	n.s.	0.18	0.076	n.s.	0.05	0.283	2.461	0.49	0.002
FeBML	4.073	0.98	< 0.001	5.549	0.36	0.015	4.096	0.66	0.004	3.670	0.56	< 0.001
TiBML	2.005	0.67	0.012	3.897	0.48	0.004	4.440	0.83	< 0.001	3.641	0.61	< 0.001
FeENT1	n.s.	0.15	0.195	n.s.	0.18	0.073	n.s.	0.14	0.160	n.s.	0.04	0.227
FeENT2	311.953	0.77	0.005	n.s.	0.06	0.206	174.299	0.81	0.000	386.603	0.22	0.038
FeENT3	n.s.	0.12	0.222	192.669	0.31	0.025	n.s.	0.20	0.117	n.s.	0.06	0.188
TiENT	n.s.	0.01	0.422	n.s.	0.25	0.101	n.s.	0.05	0.289	n.s.	0.07	0.177
FeBML												
BM	0.423	0.78	0.004	n.s.	0.04	0.262	n.s.	0.06	0.261	0.928	0.27	0.023
Stature	0.246	0.98	< 0.001	0.180	0.36	0.015	0.244	0.66	0.004	0.272	0.56	0.001
TiBML	0.472	0.78	0.004	0.776	0.77	< 0.001	1.200	0.60	0.007	1.003	0.67	< 0.001
FeENT1	n.s.	0.22	0.147	n.s.	< 0.01	0.491	n.s.	0.34	0.051	n.s.	0.03	0.266
FeENT2	76.790	0.76	0.005	n.s.	0.14	0.100	54.228	0.54	0.012	105.282	0.27	0.029
FeENT3	n.s.	0.08	0.274	n.s.	0.032	0.281	40.830	0.38	0.039	n.s.	0.08	0.156
TiENT	n.s.	< 0.01	0.459	n.s.	0.030	0.285	n.s.		0.271	0.076	0.18	0.058
TiBML												
BM	n.s.	0.70	0.009	n.s.	0.10	0.152	n.s.	< 0.01	0.473	0.724	0.48	0.002
Stature	0.499	0.67	0.012	0.257	0.480	0.004	0.225	0.83	< 0.001	0.275	0.61	< 0.001
FeBML	2.118	0.78	0.004	1.289	0.77	< 0.001	0.833	0.60	0.007	0.997	0.67	< 0.001
FeENT1	51.959	0.45	0.049	n.s.	< 0.01	0.477	n.s.	0.13	0.172	50.880	0.25	0.028
FeENT2	170.604	0.62	0.018	n.s.	0.06	0.201	n.s.	0.51	0.015	92.876	0.33	0.012
FeENT3	n.s.	0.01	0.397	n.s.	0.140	0.104	n.s.	0.27	0.075	45.383	0.25	0.029
TiENT	n.s.	< 0.01	0.497	n.s.	0.06	0.216	n.s.	0.13	0.166	36.512	0.23	0.041

*Note:* Reported are the estimated slope, *r* square, and *p*‐value for each regression.

**FIGURE 3:**
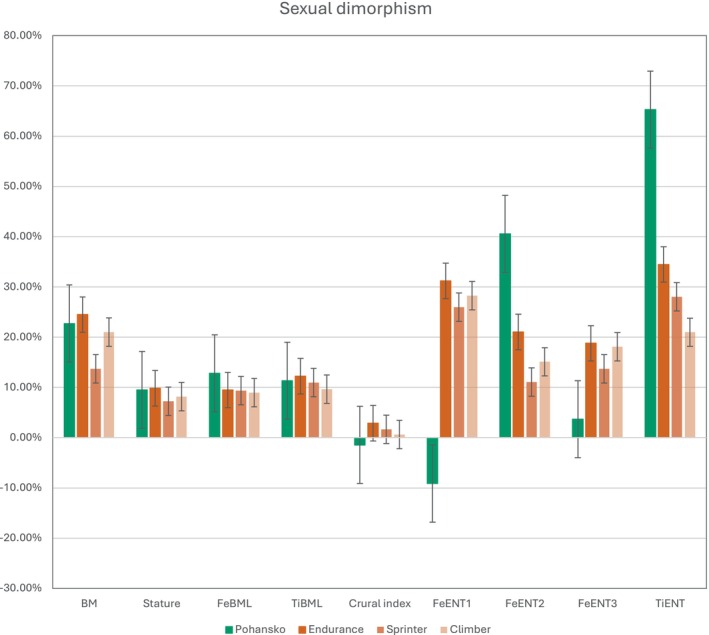
Sexual dimorphism results for BM, Stature, FeBML, TiBML, Crural Index, and unadjusted entheses among females and males belonging to the same activity sample. Positive values indicate greater male values, negative values greater female values.

Figure 3 and Table 5 summarize the extent of sexual dimorphism across body proportions and entheseal measurements. All groups exhibit positive sexual dimorphism in stature, body mass, and femoral and tibial biomechanical lengths. No significant sex differences are observed in crural index values. Unadjusted entheseal areas show contrasting patterns between the athlete and Pohansko samples: athletes display a consistent pattern of larger entheses in males across all four locations, whereas Pohansko males have smaller *gluteusmedius* (FeENT1) entheseal areas, noticeably larger *gluteus maximus* and *vasti* (FeENT2) and soleus (TiENT) entheseal areas, and comparable *gastrocnemius* (FeENT3) entheseal areas values relative to females.

**TABLE 5:** Sexual dimorphism calculation results for BM, Stature, FeBML, TiBML, Crural Index, unadjusted entheses and adjusted entheses among females and males belonging to the same Activity sample.

Sexual dimorphism	Pohansko	Endurance	Sprinter	Climber
BM	22.72%	24.49%	13.71%	21.01%
Stature	9.52%	9.83%	7.27%	8.15%
FeBML	12.82%	9.47%	9.38%	8.97%
TiBML	11.32%	12.25%	10.95%	9.62%
Crural index	−1.44%	2.87%	1.67%	0.65%
FeENT1	−9.13%	31.21%	25.98%	28.26%
FeENT2	40.56%	21.05%	11.06%	15.10%
FeENT3	3.68%	18.79%	13.71%	18.09%
TiENT	65.30%	34.48%	28.02%	20.98%

*Note:* Positive values indicate greater male values, negative values greater female values.

### 3.2 Entheses and Muscle Synergy Results

**FIGURE 4:**
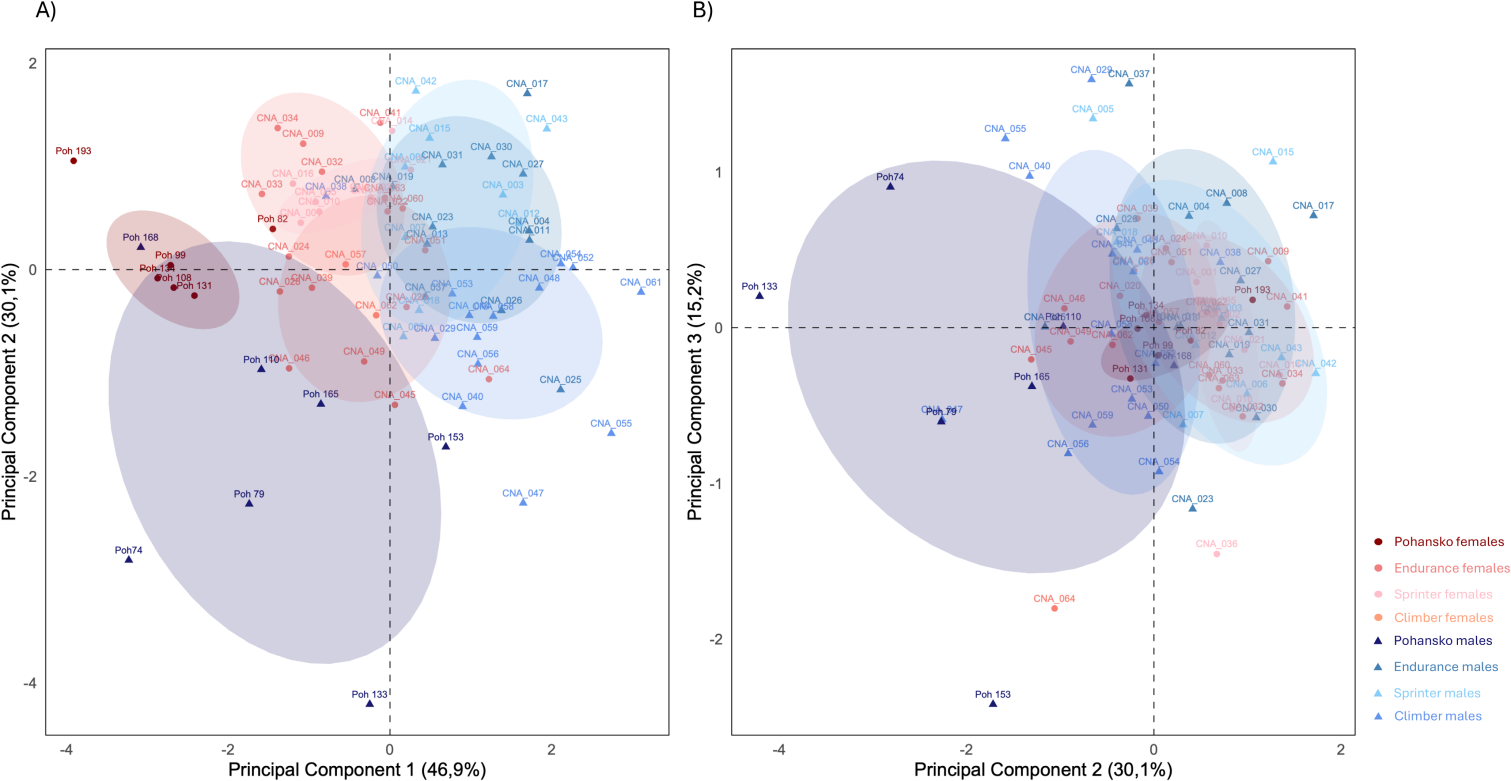
Two‐dimensional plots of the first three principal components derived from a correlation‐based principal component analysis (PCA) of the four unadjusted entheseal 3D area measurements in all 77 female (filled circles) and male (filled triangles) individuals. Ellipses represent 70% confidence intervals encompassing the distribution of individuals within each group. (A) PC1 vs. PC2; (B) PC2 vs. PC3.

Figure 4 presents the principal component analysis results for unadjusted entheses, showing that PC1 (53.1% of variance) primarily separates the Pohansko individuals from the Modern Athlete groups, and PC2 (28.4%) distinguishes both types of runners from climbers within the athletes' group and males from females within the Pohansko group. Female and male athletes cluster within their respective sex groups along PC1. Additionally, Supplementary Figure S3 displays the principal analyses results for size‐adjusted entheses when using body mass and biomechanical length, where a similar differentiation pattern is observed.

Supplementary Figure S4 and Supplementary Table S6 show the PCA based on relative entheseal proportions revealed differences across activity groups and age, with Pohansko individuals showing elevated PC1 scores driven by higher relative values of FeENT2, especially significant among Pohansko males. Relative entheseal proportions only correlated with age and activity, where age showed a significant effect on multivariate variation (*R* ^2^ = 0.066, *p* = 0.002), while activity explained a larger proportion of the variation (*R* ^2^ = 0.393, *p* = 0.001). The interaction between age and activity was not significant (*p* = 0.704). The association between age and relative entheseal proportions was mainly represented by PC1 (68.2% of variance; *r* = 0.304, *p* = 0.007), with no significant correlations observed for the remaining PCs.

**FIGURE 5:**
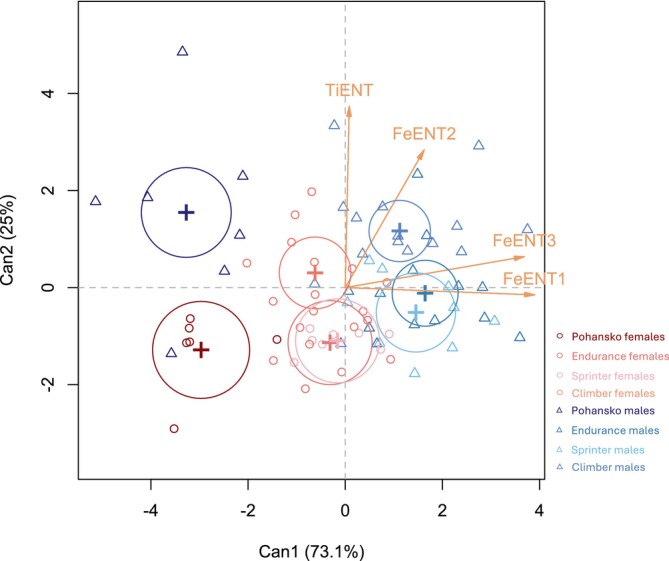
Two‐dimensional plot of the first two canonical variates (Can1 and Can2) derived from a canonical variate analysis (CVA) based on the four entheseal 3D area measurements in all 77 female (filled circles) and male (filled triangles) individuals. Ellipses represent one standard deviation (≈70% confidence interval) encompassing the distribution of individuals within each group and crosses represent the group mean.

Figure 5 shows the pooled canonical variate analysis and Tables 6, 7 display the CVA results and their standardized coefficients respectively. Can1 explains 73.37% of the variance and separates Pohansko individuals from all athlete groups, with the strongest loadings from the *gluteus medius* (FeENT1) and *gastrocnemius* (FeENT3) entheseal areas. Can2 accounts for 24.92% of the variance and separates groups based on variation in the *soleus* (TiENT) entheseal area. The distinction between male and female athletes is less pronounced than the separation between Pohansko males and females. Additionally, males and females are separated by the *soleus* (TiENT) entheseal area among Pohansko individuals while among Modern Athletes, males and females are separated by the *gluteus medius* (FeENT1), *gluteus maximus* and *vasti* (FeENT2), and *gastrocnemius* (FeENT3) entheseal areas.

**TABLE 6:** Standardized canonical coefficients derived from the canonical variate analysis (CVA) based on four entheseal 3D surface area measurements in all 77 female and male individuals.

	Can1	Can2
FeENT1	0.737	−0.233
FeENT2	−0.140	0.581
FeENT3	0.577	0.038
TiENT	0.033	0.754

**TABLE 7:** Canonical variate analyses results from the canonical variate analysis (CVA) based on four entheseal 3D surface area measurements in all 77 female and male individuals.

Can	CanRsq	Eigenvalue	Difference	Percent	Cumulative
1	0.753	3.047	2.012	73.377	73.377
2	0.508	1.035	2.012	24.915	98.292
3	0.049	0.052	2.012	1.248	99.540
4	0.019	0.019	2.012	0.460	100.000

**FIGURE 6:**
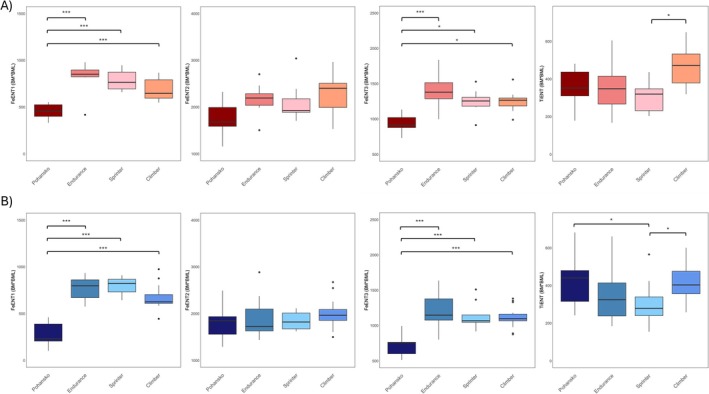
Boxplot of all female (A) and male (B) adjusted entheses. Entheses were adjusted to size using the BM and the biomechanical length of the femur and tibia. Asterisks indicate significance: *p value < 0.05; **p value < 0.01; ***p value < 0.001.

**FIGURE 7:**
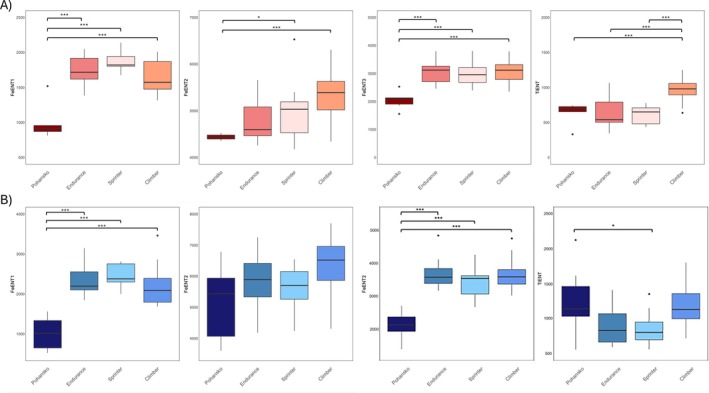
Boxplot of all female (A) and male (B) unadjusted entheses. Entheses were not adjusted to size. Asterisks indicate significance: *p value < 0.05; **p value < 0.01; ***p value < 0.001.

Tables 8 and 9 summarize adjusted entheseal areas for female and male samples. Among females, the *gluteus medius* (FeENT1) and *gastrocnemius* (FeENT3) entheseal areas show the clearest differences between the Pohansko and athlete groups (Figures 6A and 7A), with the *soleus* (TiENT) entheseal area differing significantly between sprinters and climbers. The male results follow a similar pattern: the *gluteus medius* (FeENT1) and *gastrocnemius* (FeENT3) entheseal areas differentiate Pohansko from the athlete groups, whereas the *soleus* (TiENT) entheseal area distinguishes between Pohansko and sprinters as well as sprinters and climbers (Figures 6B and 7B). For both sexes, the Pohansko *gluteus medius* (FeENT1), *gluteus maximus* and *vasti* (FeENT2) and *gastrocnemius* (FeENT3) entheses are consistently smaller than those of the athlete groups, while the *soleus* (TiENT) entheseal area does not follow this pattern.

**TABLE 8:** Descriptive statistics for the entheseal surface area measurements of all female individuals studied (number of individuals per group reported in brackets).

Entheses	Pohansko (*n* = 6)		Endurance (*n* = 8)		Sprinter (*n* = 8)		Climber (*n* = 11)		Significant diff.
	Mean	SD	Mean	SD	Mean	SD	Mean	SD	
FeENT1	996.95	261.473	1746.08	236.070	1905.09	135.907	1646.99	251.503	PF‐EF; PF‐SF; PF‐CF
FeENT2	3788.29	729.348	4679.92	572.119	5154.95	694.662	5568.33	1090.162	n.s.
FeENT3	2039.60	324.062	3036.77	452.328	2982.32	497.836	3030.81	449.781	PF‐EF; PF‐SF; PF‐CF
TiENT	639.12	155.995	619.22	253.010	630.36	143.197	959.48	178.694	PF‐CF; EF‐CF; SF‐CF
FeENT1_BM*BML	454.33	87.190	818.20	170.366	784.50	106.271	685.47	119.388	PF‐EF; PF‐SF; PF‐CF
FeENT2_BM*BML	1749.33	409.807	2165.87	350.390	2107.74	427.184	2314.76	433.420	n.s.
FeENT3_BM*BML	935.46	141.794	1407.28	276.062	1246.92	178.124	1252.32	143.442	PF‐EF; PF‐SF; PF‐CF
TiENT_BM*BML	354.21	111.247	354.14	142.824	305.43	82.870	465.57	113.739	SF‐CF

*Note:* The mean's units are the same as the ones of the reported enthesis, and they depend on the adjustment method used (unadjusted entheses in mm^2^ and BM*BML adjusted in mm/kg).

**TABLE 9:** Descriptive statistics for the entheseal surface area measurements of all male individuals studied (number of individuals per group reported in brackets).

Entheses	Pohansko (*n* = 7)		Endurance (*n* = 13)		Sprinter (*n* = 9)		Climber (*n* = 15)		Significant diff.
	Mean	SD	Mean	SD	Mean	SD	Mean	SD	
FeENT1	874.60	416.072	2391.78	443.224	2436.77	302.566	2188.91	491.251	PM‐EM; PM‐SM; PM‐CM
FeENT2	5469.41	1621.897	5781.00	897.718	5621.87	765.263	6477.90	1035.871	n.s.
FeENT3	2118.27	395.805	3666.38	456.441	3441.93	490.565	3633.44	472.252	PM‐EM; PM‐SM; PM‐CM
TiENT	1258.73	497.167	903.09	277.670	813.26	293.589	1184.36	300.386	PM‐SM; SM‐CM
FeENT1_BM*BML	283.59	134.264	775.88	115.788	803.02	89.532	671.06	130.476	PM‐EM; PM‐SM; PM‐CM
FeENT2_BM*BML	1802.31	407.204	1897.56	411.385	1846.88	186.044	1991.03	317.070	n.s.
FeENT3_BM*BML	689.24	180.253	1206.53	230.061	1137.44	184.650	1119.69	149.408	PM‐EM; PM‐SM; PM‐CM
TiENT_BM*BML	504.51	248.240	345.97	129.940	313.18	120.673	416.57	91.809	PM‐SM; SM‐CM

*Note:* The mean's units are the same as the ones of the reported enthesis, and they depend on the adjustment method used (unadjusted entheses in mm^2^ and BM*BML adjusted in mm/kg).

## 4 Discussion

Our results partly support hypothesis (1), as we observe that except for the *soleus* enthesis, Modern Athletes display significantly larger entheseal areas than the Early Medieval individuals from Pohansko. These observations are consistent with the lower limb loadings associated with athletic training, as Modern Athletes form a distinct multivariate cluster clearly separated from Pohansko individuals in the multivariate analyses. Nonetheless, our observations did not support hypothesis (2), as sexual dimorphism does not follow a uniform pattern across samples. While Modern Athletes display a consistent pattern in terms of sexual dimorphism where males present larger entheseal areas than females for all the analysed entheses, the Pohansko sample shows marked enthesis‐specific variability, with males presenting larger, comparable, or smaller entheseal areas than females depending on the muscle attachment considered.

The outcomes of the sexual dimorphism analysis show that among Modern Athletes, males present larger unadjusted entheseal areas than females for all the insertion areas of the muscles analyzed in our study (i.e., *gluteus medius*, *gluteus maximus*, *vasti*, *gastrocnemius* and *soleus*), as well as larger body mass, stature, and femoral and tibial biomechanical lengths. These results are consistent with previous research reporting greater body size, skeletal robusticity, and lower limb strength in males across Holocene populations (Berner et al. 2018; Ruff 1987). Furthermore, our multivariate analyses show a clear distinction between males and females along PC1 and Can 1. The insertion areas of the *gluteus medius* (FeENT1), *gluteus maximus* and *vasti* (FeENT2), and *gastrocnemius* (FeENT3) muscles contribute most strongly to the variance explained by PC1, whereas the *soleus* (TiENT) insertion contributes less. This pattern is consistent with previous studies showing that, even when adjusted to size, males generally present larger muscle size than females across most lower limb muscles, although the *soleus* exhibits comparatively lower levels of sexual dimorphism (Bastir et al. 2011; Olewnik et al. 2025). Accordingly, our results suggest that the *gluteus medius*, *gluteus maximus*, *vasti* and *gastrocnemius* entheseal areas contribute more strongly to sex differentiation among athletes than the *soleus* entheseal area. These findings further suggest that entheseal areas of the *gluteus medius* (FeENT1), *gluteus maximus* and *vasti* (FeENT2) and *gastrocnemius* (FeENT3) muscles may primarily reflect sex‐related differences in body size and mechanical loading more strongly than variation associated to a specific locomotor function.

Furthermore, the multivariate results show that the *soleus* entheseal (TiENT) area distinguishes climbers from both runner groups by contributing to the variance explained by PC2 and Can 2, a distinction not shared by the *gluteus maximus* (FeENT1), *gluteus medius* and *vasti* (FeENT2), or *gastrocnemius* (FeENT3) entheseal areas. Different anatomical studies (Fukutani et al. 2020; Gollnick et al. 1974; Kapoor et al. 2025; Lenhart et al. 2014; Olewnik et al. 2025) have shown that the *soleus* muscle plays a fundamental role in postural control, gait mechanics, and plantarflexion, particularly during activities involving knee flexion (Ward et al. 2009). The *soleus* is essential for sustaining the tonic contractions required to maintain upright posture and enable energy‐efficient locomotion. During quiet standing, it functions as a primary antigravity muscle, counteracting anterior sway by generating continuous, low‐level plantarflexion torque at the ankle (Ward et al. 2009). In fact, the muscular pattern of the *soleus* is primarily interpreted as activity for support rather than for propulsion (Hanna and Schmitt 2011; Olewnik et al. 2025). Additionally, the activation of the *soleus* and the *gastrocnemius* depends on the speed of locomotion, with their early activation only happening at higher speeds (Arnold et al. 2013; Hanna and Schmitt 2011). Running is characterized by highly cyclical sagittal‐plane locomotion with repetitive, high‐magnitude ground‐reaction forces (Hernández‐Stender et al. 2024; Novacheck 1998) while climbing involves non‐cyclical, task‐dependent movements in which the lower limb contributes primarily to stabilization (Faggian et al. 2025; García‐Heras et al. 2025; Quaine and Martin 1999). The specific biomechanical demands of running and climbing in conjunction with the specific characteristics of the *soleus* contribute to explaining the differentiation among climbers and both groups of runners due to the different use of the muscle during their activities: climbers use the *soleus* for stabilization, while in both groups of runners it also seems to contribute to the production of propulsive force (Arnold et al. 2013; Hanna and Schmitt 2011). Thus, the *soleus* (TiENT) entheseal area is consistent with a functional adaptation to different mechanical requirements, suggesting that its entheseal area may reflect functional variation in stabilization versus propulsion allowing to distinguish locomotor strategies based on the muscle's sensitivity to the specialized stabilization demands of climbing compared with the cyclical, high‐magnitude loading of running.

Though climbers can be clearly distinguished from both runner groups, our results show that in the univariate and multivariate analyses sprinters and endurance runners present a large overlap, showing no significant differences across any of the entheseal areas analyzed in this study. Previous research established clear physiological differences at the muscular level among sprinter and endurance runners (Abe et al. 2000; Bex et al. 2017; Crowther et al. 2002; Fukutani et al. 2020; Hennessy 2017; Hiikkinen and Keskinen 1989; Kusy and Zieliński 2015; Maughan et al. 1983; Picincu and Braveman 2020). Sprinters exhibit significantly greater lower‐limb muscle volume, cross‐sectional area and thickness along with a higher proportion and larger size of type II fibers, greater anaerobic enzyme activity, and superior maximal force and power production, whereas endurance runners display smaller muscle size, a predominance of type I fibers, higher capillary density, and enhanced oxidative capacity supporting sustained submaximal force and fatigue resistance (Abe et al. 2000; Bex et al. 2017; Crowther et al. 2002; Fukutani et al. 2020; Hennessy 2017; Hiikkinen and Keskinen 1989; Kusy and Zieliński 2015; Maughan et al. 1983; Picincu and Braveman 2020). Though sprinters and endurance runners do present significant muscular physiological differences, our results do not seem to capture such diversity, suggesting that entheseal areas might not be sensitive to muscle physiological or microstructural differences.

When comparing the Pohansko individuals to Modern Athletes, it is possible to observe substantial differences within the patterns resulting from analyzing their entheseal areas. Modern Athletes present larger entheseal areas than Pohansko individuals for all the muscles analyzed in this study. This result supports that entheseal areas are the product of cumulative mechanical loadings (Benjamin et al. 2006; Karakostis, Jeffery, and Harvati 2019; Karakostis, Wallace, et al. 2019; Villotte et al. 2010). Moreover, while Modern Athlete males present larger unadjusted entheseal areas than Modern Athlete females for all the muscle insertions analyzed in our study, Pohansko males only have significantly larger unadjusted *gluteus maximus* and *vasti* (FeENT2) and *soleus* (TiENT) entheseal areas, while comparable *gastrocnemius* (FeENT3) and significantly smaller *gluteus medius* (FeENT1) entheseal areas than Pohansko females. Our observations regarding the Pohansko sample are consistent with previous studies of Early Medieval populations that found that females exhibited larger *gluteus medius* (FeENT1) entheseal areas than males, implying sex‐specific muscle use rather than simple strength‐related differences (Havelková et al. 2011). Moreover, our results also show that Pohansko males exhibit *soleus* entheseal area values that overlap more closely with the range observed among climbers, while Pohansko females show values more similar to both groups of runners. Comparable associations have been reported in archaeological populations, where male individuals engaged in activities such as horse riding display relatively larger *soleus* (TiENT) entheseal areas (Bühler and Kirchengast 2022; Djukic et al. 2018). Together with archaeological evidence from Pohansko, where some males were buried with horse‐related artifacts, these results suggest that variation in soleus entheseal area may reflect sex‐based differences in lower limb loading and posture associated with habitual activities, potentially including horse riding among males. Consequently, this interpretation is more likely associated with behavioral differentiation among Pohansko males and females rather than differences attributable solely to overall strength, being consistent with previous results of sex‐based division of labour in Early Medieval populations (Foster et al. 2014; Havelková et al. 2013; Laffranchi et al. 2020). This interpretation is further supported by the results of relative entheseal proportions, which showed that Pohansko individuals, particularly males, exhibited a proportionally greater *gluteus maximus* and *vasti* (FeENT2) entheseal areas relative to the other muscle insertions than the Modern Athlete groups. As these muscles are the principal extensors of the hip and knee (Al‐Oumaoui et al. 2004; Lieverse et al. 2013; Robb 1994; Takigawa 2014), this pattern is consistent with a greater relative emphasis on lower limb extension associated with sustained weight‐bearing activities, such as load carrying, repeated squatting or rising (Goldberg and Kepple 2009; Maniar et al. 2022; Robertson et al. 2008; Sugisaki et al. 2014), rather than the highly specialized locomotor demands of Modern Athletes. Because these results are based on relative entheseal proportions, they complement the entheseal area results by establishing differences in the distribution, rather than the overall magnitude, of entheseal development. Altogether these patterns may reflect differences not only in activity type but also in loading regime, with Modern Athletes representing high‐intensity, specialized loading and Pohansko individuals reflecting moderate but sustained habitual activity. This distinction may underlie the contrasting entheseal patterns observed between samples.

Hence, analyzing the entheseal areas of the Modern Athletes sample allowed us to better characterize the relationship between activity and entheseal morphology when controlling for age, sex, and body size. By doing so, we established an activity‐based comparative framework that helped to better contextualize the results of the Pohansko sample. Using data from athletes and sports remains one of the best approaches to infer and reconstruct behavioural patterns from past populations as comparative modern samples provide a useful biomechanical context (Longman et al. 2020; Milks et al. 2019; Shaw and Stock 2013). However, the absence of a modern non‐athlete control group limits the interpretation of the observed differences between Modern Athletes and Pohansko individuals, as these may reflect not only athletic loading but also factors such as body size, secular changes, nutrition, or sample composition. While athlete samples provide a valuable model of well‐defined and intense mechanical loading, they do not capture the full range of variability present in non‐specialized, everyday activity patterns. As a result, comparisons between these populations should be interpreted cautiously, refraining from extending these results beyond broad functional similarities. Additionally, age also remains a limitation and could be the reason for the overlap between sprinters and endurance runners as sprinters are significantly younger than endurance runners in our sample. Although previous studies have reported correlations between entheseal areas and age (Milella et al. 2012; Sick 2021; Villotte et al. 2010; Villotte and Santos 2023), this relationship is not consistently observed (Bousquié et al. 2022; Godde et al. 2018; Karakostis, Jeffery, and Harvati 2019). This discrepancy might be linked to the fact that individuals from the same population perform different activities and thus present different entheseal area patterns. In our sample, one possible explanation for the overlap between sprinters and endurance runners is that the higher mechanical intensity associated with sprinting may exert a stronger influence on entheseal development than the lower intensity but repetitive loading characteristic of endurance running, potentially masking age‐related effects. Nonetheless, relative entheseal proportions showed a significant association with age, though this effect was modest and considerably weaker than that of activity. The lack of an age‐by‐activity interaction indicates that the differences between the Pohansko and Modern Athlete groups are more likely to reflect variation in habitual loading than age‐related changes. Future research should further investigate the interaction between age and activity‐related loading to better understand their respective contributions to entheseal variation and improve the reconstruction of activity patterns in past populations.

## 5 Conclusion

The comparison of the entheseal areas' size of our Modern Athletes and Pohansko samples allows for five general conclusions:

1. The *gluteus maximus*, *gluteus medius*, *vasti*, and *gastrocnemius* entheseal areas suggest a stronger association with load magnitude than with activity differentiation under the conditions tested, reflecting sex‐related differences in body size among Modern Athlete females and males.
2. The *soleus* entheseal area shows a larger sensitivity to locomotor patterns than to sex differences in Modern Athletes, contributing to distinguish between climbers and both groups of runners.
3. Entheseal areas do not seem to be sensitive to muscle physiological or microstructural differences that could differentiate among sprinters and endurance runners.
4. Pohansko individuals present a contrasting pattern to the one observed in Modern Athletes, consistent with broader patterns of reduced sexual dimorphism documented in sedentary Holocene populations and distinct female and male behaviors as described previously for Early Medieval populations.
5. It is crucial to use a well‐known comparative sample when analyzing entheses from past populations to provide an appropriate framework for interpreting past population entheseal areas.

## Author Contributions

**Elena García‐Vargas:** conceptualization, methodology, data curation, investigation, validation, formal analysis, visualization, project administration, writing – original draft, writing – review and editing, software. **Enrique Alcalá:** writing – review and editing, data curation. **Jiří Macháček:** writing – review and editing, resources. **Vladimír Sládek:** data curation, conceptualization, validation, supervision, funding acquisition, resources, writing – review and editing. **Markus Bastir:** data curation, resources, funding acquisition, writing – review and editing. **Víctor Manuel Encinas‐Tobajas:** methodology, data curation, writing – review and editing. **Ángel Luis Parrado‐Gallego:** data curation, methodology, writing – review and editing.

## Funding

This work was supported by the EXPRO project 21‐17092X (GAČR) “The Formation of Multi‐ethnic Complex Societies in Early Medieval Moravia: Collective Action Theory and an Interdisciplinary Approach” awarded to V. Sládek; by project PID2020‐115854GB‐I00 awarded to M. Bastir, which supported CT data collection; and by the project “Ready for the Future: Understanding Long‐Term Resilience of Human Culture” (RES‐HUM; CZ.02.01.01/00/22_008/0004593) funded by the Czech Ministry of Education, Youth and Sports and awarded to J. Macháček.

## Ethics Statement

All procedures involving human participants were reviewed and approved by the Ethics Committee of the Faculty of Science, Charles University (approval number 2024/03) and by the Scientific Commission of the Centro Nacional de Aceleradores (Seville), where the study was conducted. All procedures were performed in accordance with the ethical standards of the responsible institutional committees and with the 1964 Helsinki Declaration and its later amendments. Prior to participation, all volunteers were fully informed about the study objectives, procedures, and potential risks, and provided written informed consent. All data were anonymised prior to acquisition, processing, and analysis. Research on human skeletal remains was conducted in accordance with relevant institutional, national, and international guidelines, with permission granted by the appropriate curating institutions.

## Conflicts of Interest

The authors declare no conflicts of interest.

## Supporting information


**Supporting Information S1:** Supporting Information.

**Figure S1:** Two‐dimensional plots of the first three principal components derived from a correlation‐based principal component analysis (PCA) of the four adjusted to size (using the body mass) entheseal 3D area measurements in all 77 female and male individuals. Ellipses represent 70% confidence intervals encompassing the distribution of individuals within each group. (A) PC1 vs. PC2; (B) PC2 vs. PC3.

**Figure S2:** Boxplot of all female (A) and male (B) adjusted to size entheses. Entheses were adjusted to size using body mass. Asterisks indicate significance: *p value < 0.05; **p value < 0.01; ***p value < 0.001.

**Figure S3:** Two‐dimensional plots of the first three principal components derived from a correlation‐based principal component analysis (PCA) of the four adjusted to size (using the body mass and biomechanical length) entheseal 3D area measurements in all 77 female and male individuals. Ellipses represent 70% confidence intervals encompassing the distribution of individuals within each group. (A) PC1 vs. PC2; (B) PC2 vs. PC3.

**Figure S4:** Two‐dimensional plots of the first three principal components derived from a correlation‐based principal component analysis (PCA) of the relative entheseal proportions in all 77 female and male individuals. Ellipses represent 70% confidence intervals encompassing the distribution of individuals within each group. (A) PC1 vs. PC2; (B) PC2 vs. PC3.

**Figure S5:** Boxplot of all female (A) and male (B) relative entheseal proportions for PC1, the only principal component that presented significant differences among groups. Asterisks indicate significance: *p ≤ 0.05; **p ≤ 0.01; ***p ≤ 0.001.

**Table S1:** Significant differences among samples with sexes pooled.

**Table S2:** Characteristics of each individual in the Modern Athlete sample, including activity, sex, age, years of practice, training frequency (daily and weekly), and previous activity (if applicable). Participants CNA_026 and CNA_055 did not provide survey responses prior to publication.

**Table S3:** Statistics of the principal component analysis (correlation matrix) on the 3D surface measurements of the three femoral entheses and the one tibial enthesis analyzed when not adjusted to size of all 77 female and male individuals.

**Table S4:** Statistics of the principal component analysis (correlation matrix) on the 3D surface measurements of the three femoral entheses and the one tibial enthesis when adjusted to size using body mass for all 77 female and male individuals.

**Table S5:** Statistics of the principal component analysis (correlation matrix) on the 3D surface measurements of the three femoral entheses and the one tibial enthesis when adjusted to size using body mass and biomechanical length for all 77 female and male individuals.

**Table S6:** Statistics of the principal component analysis (correlation matrix) on the 3D surface measurements of the three femoral entheses and the one tibial enthesis when adjusted to size using body mass and biomechanical length for all 77 female and male individuals.

## Data Availability

The data that support the findings of this study are openly available as part of the Supporting Information accompanying this article.
