# Supplementary material for: Lower Limb Entheseal Variation in Modern Athletes and an Early Medieval Population: Implications for Activity Reconstruction
Source: Am J Biol Anthropol. 2026 Aug 18;190(4):e70335. doi: 10.1002/ajpa.70335 (PMC13482320; doi:10.1002/ajpa.70335)
Supplement: Supplementary file 1 [file AJPA-190-e70335-s001.pdf]

	PC1	PC2	PC3
FeENT1	-0.588	0.319	0.405
FeENT2	-0.488	-0.474	-0.694
FeENT3	-0.644	0.119	0.120
TiENT	-0.040	-0.812	0.582
Standard deviation	1.397	1.123	0.698
Proportion of Variance	0.488	0.315	0.122
Cumulative Proportion	0.488	0.803	0.925
