# Supplementary material for: Lower Limb Entheseal Variation in Modern Athletes and an Early Medieval Population: Implications for Activity Reconstruction
Source: Am J Biol Anthropol. 2026 Aug 18;190(4):e70335. doi: 10.1002/ajpa.70335 (PMC13482320; doi:10.1002/ajpa.70335)
Supplement: Supplementary file 2 [file AJPA-190-e70335-s002.pdf]

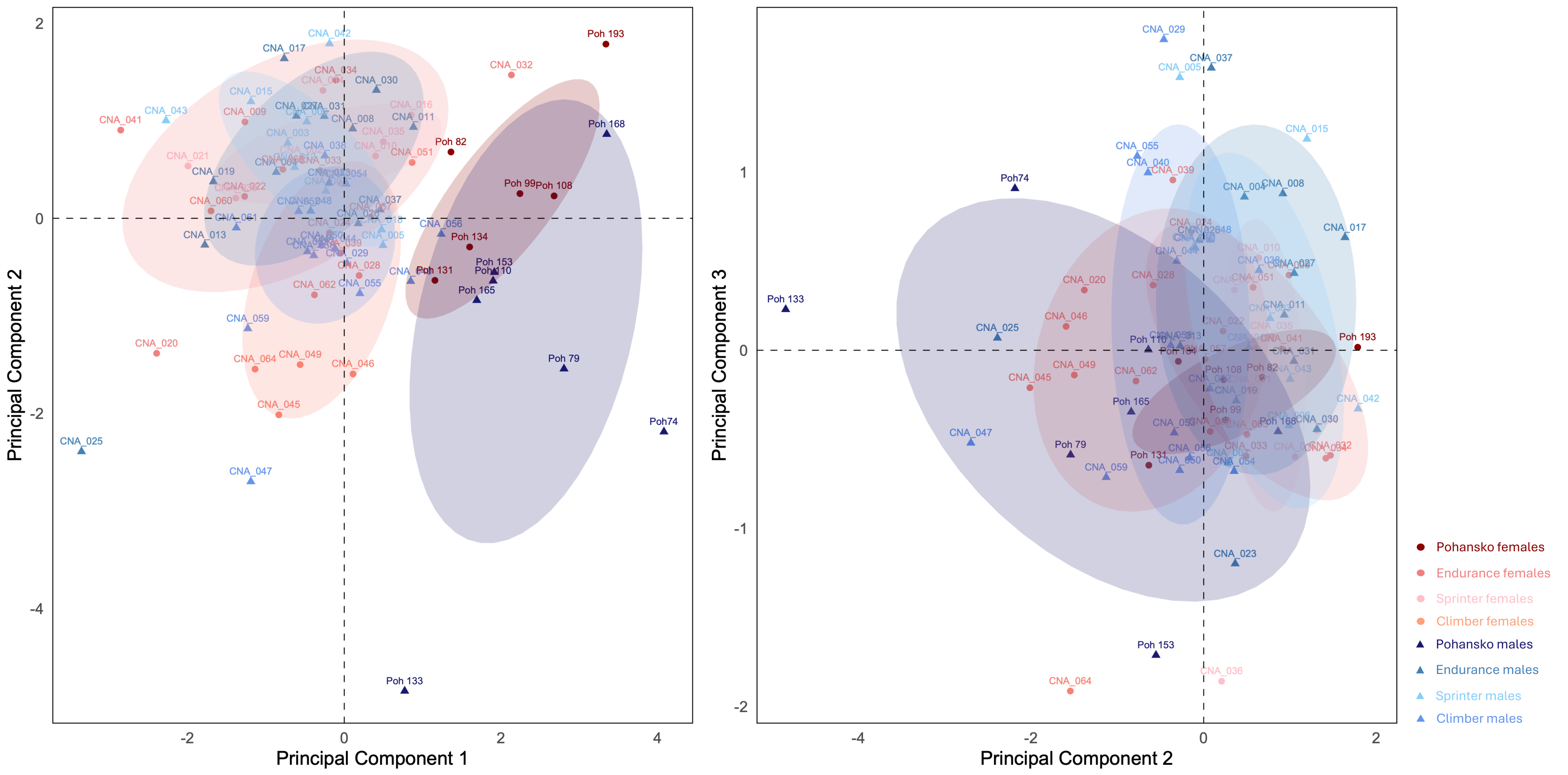

Principal Component 1
Principal Component 2
Principal Component 3
-4
-2
0
2
4
Pohansko females
Endurance females
Sprinter females
Climber females
Pohansko males
Endurance males
Sprinter males
Climber males
