# Supplementary material for: Lower Limb Entheseal Variation in Modern Athletes and an Early Medieval Population: Implications for Activity Reconstruction
Source: Am J Biol Anthropol. 2026 Aug 18;190(4):e70335. doi: 10.1002/ajpa.70335 (PMC13482320; doi:10.1002/ajpa.70335)
Supplement: Supplementary file 3 [file AJPA-190-e70335-s003.pdf]

ID	Activity	Sex	Age	Years practicing activity	Recurrence per day	Recurrence per week	Other activities	Which other activities
CNA_001	Sprinter	Female	21	13	1		Yes	Judo (3 years) y skating (1 year)
CNA_002	Sprinter	Female	22	16	1		No	
CNA_003	Sprinter	Male	20	10	1	6	Yes	Swimming (5 years) taikwondo (10 years)
CNA_004	Endurance	Male	20	9	1		No	
CNA_005	Sprinter	Male	19	13	1	4	No	
CNA_006	Sprinter	Male	19	5	1		Yes	Football (8 years)
CNA_007	Sprinter	Male	19	4	1		No	
CNA_008	Endurance	Male	27	5	1		Yes	Triathlon
CNA_009	Endurance	Female	32	15	1	4	Yes	Swiimming (14 years)
CNA_010	Sprinter	Female	25	15	1		No	
CNA_011	Endurance	Male	45	37	1		No	
CNA_012	Sprinter	Male	26	12	1		Yes	Basketball (6 years)
CNA_013	Endurance	Male	30	13	1	6	Yes	Rugby (2 years), swimming (10 years), waterpolo (1 year)
CNA_014	Sprinter	Female	22	19	1		Yes	Swimming (12 years)
CNA_015	Sprinter	Male	20	13	1	5	Yes	Rowing (1 year)
CNA_016	Sprinter	Female	22	17	2		Yes	Triple jump
CNA_017	Endurance	Male	27	7	1	6	Yes	Football (2 years)
CNA_018	Sprinter	Male	24	13	2		Yes	Karate
CNA_019	Endurance	Male	30	3	1		Yes	Rugby (2 years), swimming (7 years), Kick Boxing (2 years)
CNA_020	Endurance	Female	31	25	2	3	No	
CNA_021	Sprinter	Female	19	11	1	3	Yes	Cross and endurance running (2 years), mid-length running 600m and 800 (2 years)
CNA_022	Endurance	Female	37	27	1		Yes	Climbing and mountaineering
CNA_023	Endurance	Male	24	8	2		No	
CNA_024	Endurance	Female	42	12	1		Yes	Swimming (10 years)
CNA_025	Endurance	Male	43	30	1	6	Yes	Handball and football
CNA_026	Endurance	Male	41					
CNA_027	Endurance	Male	31	10	2		Yes	Triathlon
CNA_028	Climber	Female	28	6	1	4	Yes	Ballet (7 years)
CNA_029	Climber	Male	31	10	1		Yes	Basketball (3 years), swimming (10 years), athletism (4 years)
CNA_030	Endurance	Male	40	5	1		Yes	High level athletism
CNA_031	Endurance	Male	40	33	1	6	Yes	Badminton, football
CNA_032	Endurance	Female	44	19	1		No	
CNA_033	Endurance	Female	41	20	1		No	
CNA_034	Endurance	Female	43	12	1		Yes	Yoga
CNA_035	Sprinter	Female	32	19	1		Yes	Artistic gymnastics (4 years)
CNA_036	Sprinter	Female	30	25	1		No	
CNA_037	Endurance	Male	45	10	2		No	
CNA_038	Climber	Male	41	26	1	5	Yes	Handball (11 years), surf (20 years)
CNA_039	Climber	Female	39	1	1		Yes	Hapkido (8 years), swimming (5 years)
CNA_040	Climber	Male	19	12	2		Yes	Running (2 to 4 hours daily)
CNA_041	Endurance	Female	37	7	1	5	Yes	Running, horse riding, swimming
CNA_042	Sprinter	Male	19	14	1	6	No	
CNA_043	Sprinter	Male	18	8	1		No	
CNA_044	Climber	Male	40	25	1	2	Yes	Canyoning (since a teenager)
CNA_045	Climber	Female	23	2	1	3	Yes	Combat sports (muay Thai)
CNA_046	Climber	Female	33					
CNA_047	Climber	Male	33	10	1		Yes	Cycing (5 years)
CNA_048	Climber	Male	31	1	1		Yes	Gym, swimming, mid-distance running (last 7-8 years), preparation to become a firefighter
CNA_049	Climber	Female	36	17			Yes	High level wimming (local competitions), running and basketball (short periods of time)
CNA_050	Climber	Male	33	3	1		Yes	Athletism (1 year), swimming (1 year)
CNA_051	Climber	Female	27	15	2		Yes	Swimming (4 years), synchronised swimming (4 years)
CNA_052	Climber	Male	36	5	1	6	Yes	Swimming, cycling, running, strength exercises, football (all of it for 10 years and previous to climbing)
CNA_053	Climber	Male	39	20	1		Yes	Cycling, duathlon, triathlon, adventure raid, trail running
CNA_054	Climber	Male	33	7	1	4	No	
CNA_055	Climber	Male	31					
CNA_056	Climber	Male	45	25	1		Yes	Skiing (15 years), trail running (2 years), mountaineering (6 years)
CNA_057	Climber	Female	27	2	1	3	Yes	Yoga
CNA_058	Climber	Male	24	20	2		Yes	Tennis, mountain cycling, rugby, swimming, calisthenics, snowboard, skate, trail running
CNA_059	Climber	Male	39	30	1		Yes	Cycling (2 years), gym (always), football (many years)
CNA_060	Climber	Female	31	4	1		Yes	Rugby (9 years)
CNA_061	Climber	Male	34	3	1		Yes	High combat sports (14 years, national and international competitions)
CNA_062	Climber	Female	19	4	1	4	Yes	Artistic gymnastics (3 years)
CNA_063	Climber	Female	41	3	1		Yes	Urban skating, belly dancing, mountaineeringPatinaje urbano, danza del vientre, alpinismo
CNA_064	Climber	Female	38	5	1		Yes	High level rowing (part of the national team)
