# Supplementary material for: Lower Limb Entheseal Variation in Modern Athletes and an Early Medieval Population: Implications for Activity Reconstruction
Source: Am J Biol Anthropol. 2026 Aug 18;190(4):e70335. doi: 10.1002/ajpa.70335 (PMC13482320; doi:10.1002/ajpa.70335)
Supplement: Supplementary file 4 [file AJPA-190-e70335-s004.pdf]

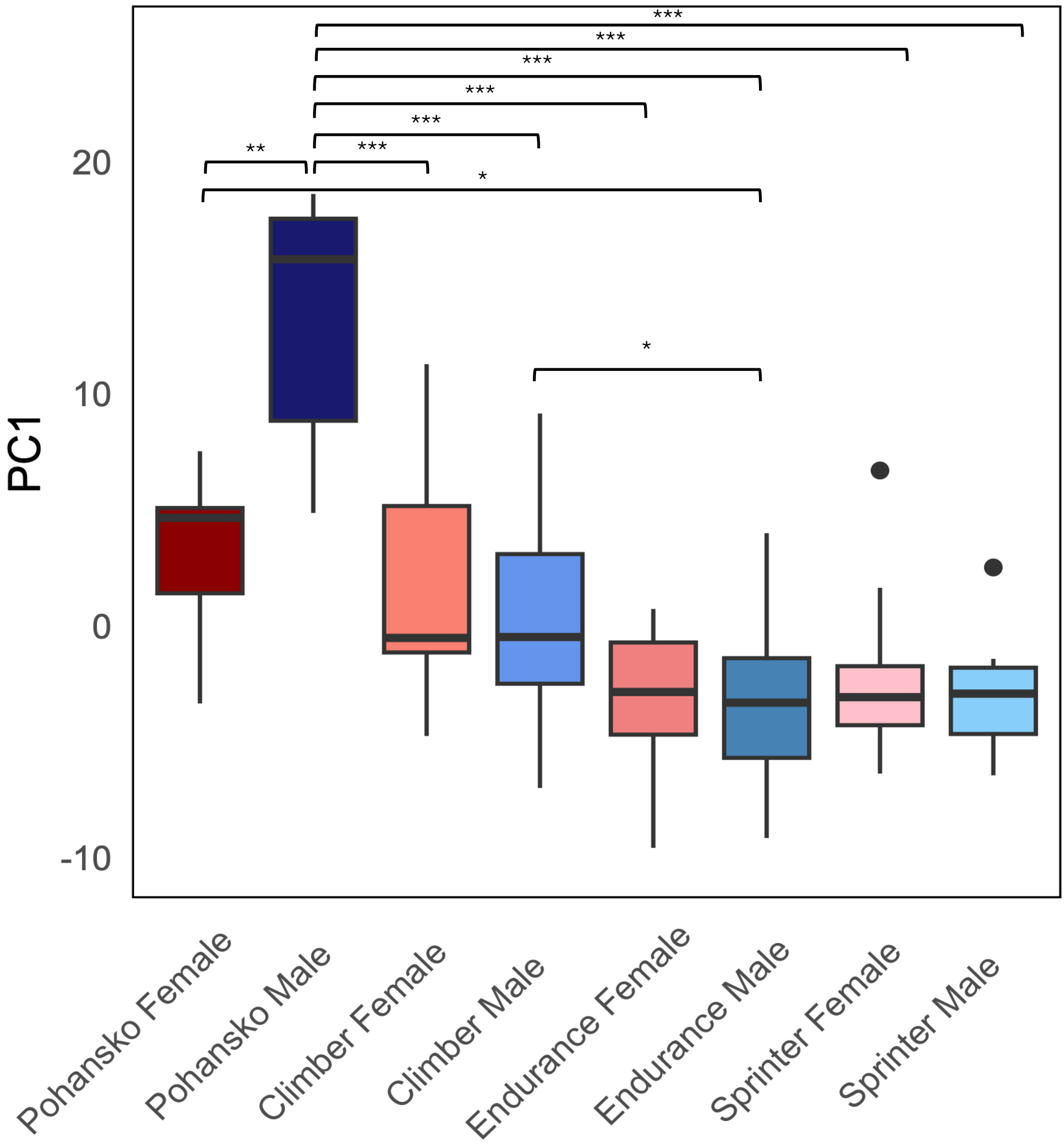

PC1
20
10
0
-10
***
***
***
***
***
***
**
*
*
Pohansko Female
Pohansko Male
Climber Female
Climber Male
Endurance Female
Endurance Male
Sprinter Female
Sprinter Male
