# Supplementary material for: Lower Limb Entheseal Variation in Modern Athletes and an Early Medieval Population: Implications for Activity Reconstruction
Source: Am J Biol Anthropol. 2026 Aug 18;190(4):e70335. doi: 10.1002/ajpa.70335 (PMC13482320; doi:10.1002/ajpa.70335)
Supplement: Supplementary file 5 [file AJPA-190-e70335-s005.pdf]

	PC1	PC2	PC3
FeENT1	0.562	0.370	0.332
FeENT2	0.529	-0.344	-0.765
FeENT3	0.593	0.274	0.163
TiENT	0.230	-0.818	0.527
Standard deviation	1.458	1.066	0.675
Proportion of Variance	0.531	0.284	0.114
Cumulative Proportion	0.531	0.815	0.929
