# Supplementary material for: Lower Limb Entheseal Variation in Modern Athletes and an Early Medieval Population: Implications for Activity Reconstruction
Source: Am J Biol Anthropol. 2026 Aug 18;190(4):e70335. doi: 10.1002/ajpa.70335 (PMC13482320; doi:10.1002/ajpa.70335)
Supplement: Supplementary file 6 [file AJPA-190-e70335-s006.pdf]

A)

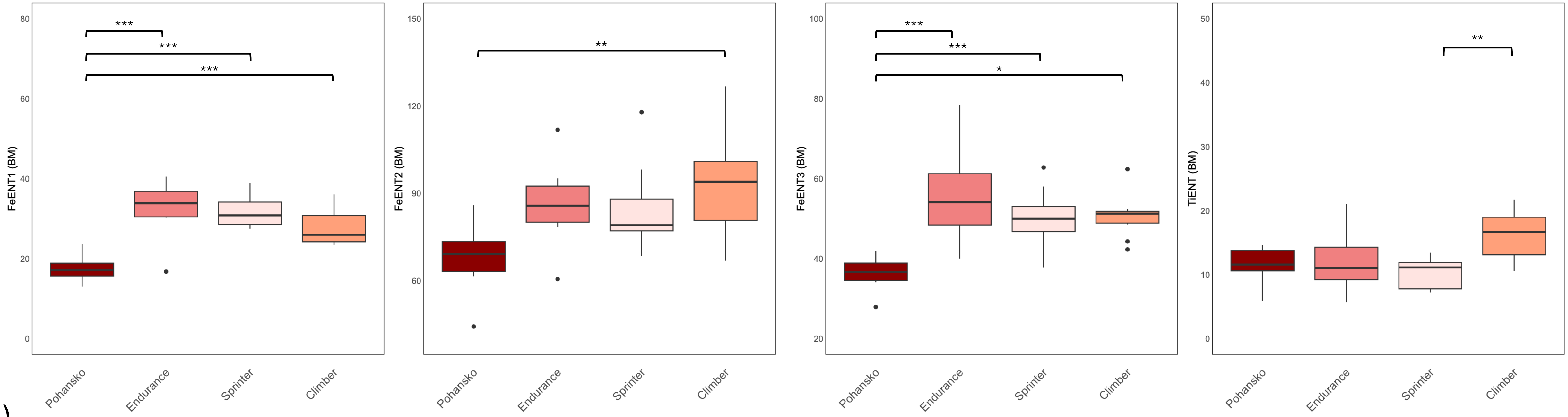
FeENT1 (BM)
FeENT2 (BM)
FeENT3 (BM)
TiENT (BM)
***
**
*
Pohansko
Endurance
Sprinter
Climber

B)

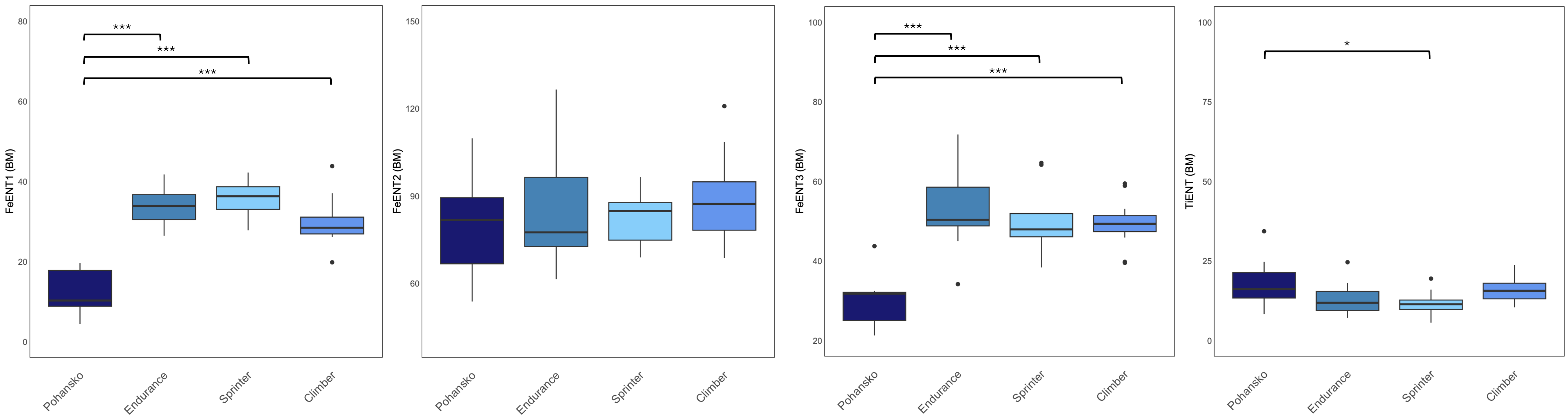
FeENT1 (BM)
FeENT2 (BM)
FeENT3 (BM)
TiENT (BM)
***
*
Pohansko
Endurance
Sprinter
Climber
