# Supplementary material for: Lower Limb Entheseal Variation in Modern Athletes and an Early Medieval Population: Implications for Activity Reconstruction
Source: Am J Biol Anthropol. 2026 Aug 18;190(4):e70335. doi: 10.1002/ajpa.70335 (PMC13482320; doi:10.1002/ajpa.70335)
Supplement: Supplementary file 7 [file AJPA-190-e70335-s007.pdf]

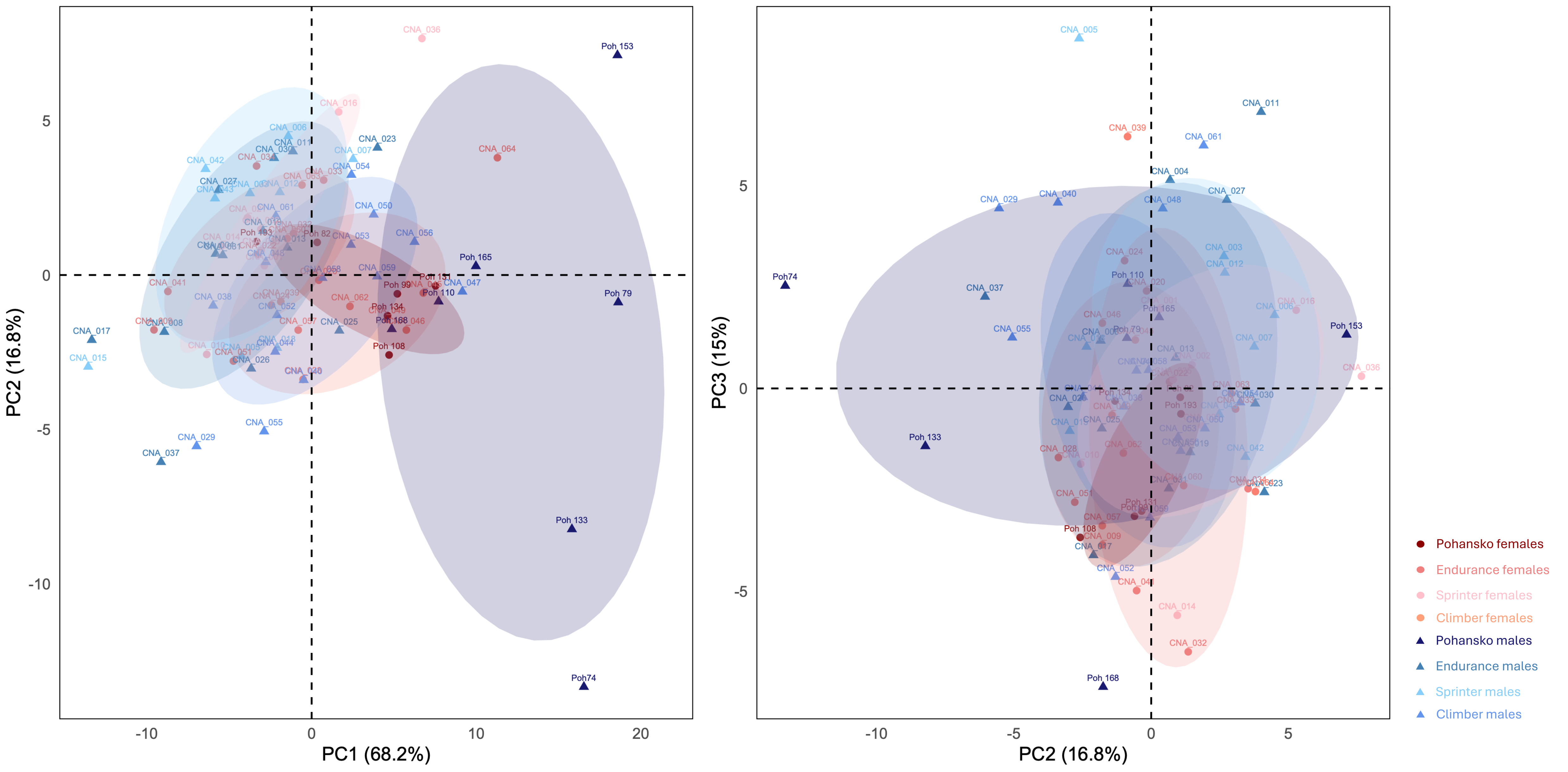

PC1 (68.2%)
PC2 (16.8%)
-10
0
10
20
5
0
-5
-10
PC2 (16.8%)
PC3 (15%)
-10
-5
0
5
5
0
-5
Pohansko females
Endurance females
Sprinter females
Climber females
Pohansko males
Endurance males
Sprinter males
Climber males
