# Supplementary material for: Lower Limb Entheseal Variation in Modern Athletes and an Early Medieval Population: Implications for Activity Reconstruction
Source: Am J Biol Anthropol. 2026 Aug 18;190(4):e70335. doi: 10.1002/ajpa.70335 (PMC13482320; doi:10.1002/ajpa.70335)
Supplement: Supplementary file 8 [file AJPA-190-e70335-s008.pdf]

	PC1	PC2	PC3
FeENT1	-0.576	0.340	0.449
FeENT2	-0.508	-0.448	-0.681
FeENT3	-0.640	0.105	0.098
TiENT	-0.043	-0.821	0.570
Standard deviation	1.414	1.123	0.672
Proportion of Variance	0.500	0.315	0.113
Cumulative Proportion	0.500	0.816	0.929
