# Supplementary material for: Lower Limb Entheseal Variation in Modern Athletes and an Early Medieval Population: Implications for Activity Reconstruction
Source: Am J Biol Anthropol. 2026 Aug 18;190(4):e70335. doi: 10.1002/ajpa.70335 (PMC13482320; doi:10.1002/ajpa.70335)
Supplement: Supplementary file 10 [file AJPA-190-e70335-s010.pdf]

Values	Pooled
	Significant diff.
Age (in years)	SF-EF : SM-EF ; SM-PF ; SM-CM ; SM-EM ; SM-PM
Body Mass (BM)(in kg)	CM-CF ; CM-EF ; EM-EF ; PM-EF ; CM-PF ; EM-PF ; CM-SF
Stature (in cm)	CM-CF ; EM-CF ; SM-CF ;CM-EF ; EM-EF ; SM-EF ; CM-PF ; EM;PF ; SM-PF ; CM-SF ; EM-SF ; SM-SF
BMI (in kg/m2)	n.s.
Femur biomechanical length (FeBML) (in	SM-CF ; CM-EF ; EM-EF ; PM-EF ; SM-EF ; CM-PF ; EM-PF ; PM-PF ; SM-PF ; CM-SF ; EM-SF ; SM-SF
Tibia biomechanical length (TiBML) (in m	CM-CF ; EM-CF ; SM-CF ; CM-EF ; SM-EF ; CM-PF ; PM-PF ; SM-PF ; CM-SF ; EM-SF ; SM-SF
Crural index	PM-CF ; PM-CM; PM-EM
