# Supplementary material for: Lower Limb Entheseal Variation in Modern Athletes and an Early Medieval Population: Implications for Activity Reconstruction
Source: Am J Biol Anthropol. 2026 Aug 18;190(4):e70335. doi: 10.1002/ajpa.70335 (PMC13482320; doi:10.1002/ajpa.70335)
Supplement: Supplementary file 11 [file AJPA-190-e70335-s011.pdf]

	PC1	PC2	PC3
FeENT1	-0.483	0.428	0.578
FeENT2	0.712	0.460	-0.178
FeENT3	-0.457	-0.118	-0.726
TiENT	0.227	-0.769	0.326
Standard deviation	6.522	3.237	3.053
Proportion of Variance	0.682	0.168	0.150
Cumulative Proportion	0.682	0.851	1.000
